# Bioinformatics analysis and consistency verification of a novel tuberculosis vaccine candidate HP13138PB

**DOI:** 10.3389/fimmu.2023.1102578

**Published:** 2023-01-27

**Authors:** Peng Cheng, Fan Jiang, Guiyuan Wang, Jie Wang, Yong Xue, Liang Wang, Wenping Gong

**Affiliations:** ^1^ Tuberculosis Prevention and Control Key Laboratory/Beijing Key Laboratory of New Techniques of Tuberculosis Diagnosis and Treatment, Senior Department of Tuberculosis, The Eighth Medical Center of PLA General Hospital, Beijing, China; ^2^ Department of Geriatrics, The Eighth Medical Center of PLA General Hospital, Beijing, China; ^3^ The Second Brigade of Cadet, Basic Medical School, Air Force Military Medical University, Xi’an, Shaanxi, China; ^4^ Hebei North University, Zhangjiakou, Hebei, China

**Keywords:** tuberculosis, epitope vaccines, immunoinformatics, immune responses, bioinformatics

## Abstract

**Background:**

With the increasing incidence of tuberculosis (TB) and the shortcomings of existing TB vaccines to prevent TB in adults, new TB vaccines need to be developed to address the complex TB epidemic.

**Method:**

The dominant epitopes were screened from antigens to construct a novel epitope vaccine termed HP13138PB. The immune properties, structure, and function of HP13138PB were predicted and analyzed with bioinformatics and immunoinformatics. Then, the immune responses induced by the HP13138PB were confirmed by enzyme-linked immunospot assay (ELISPOT) and Th1/Th2/Th17 multi-cytokine detection kit.

**Result:**

The HP13138PB vaccine consisted of 13 helper T lymphocytes (HTL) epitopes, 13 cytotoxic T lymphocytes (CTL) epitopes, and 8 B-cell epitopes. It was found that the antigenicity, immunogenicity, and solubility index of the HP13138PB vaccine were 0.87, 2.79, and 0.55, respectively. The secondary structure prediction indicated that the HP13138PB vaccine had 31% of α-helix, 11% of β-strand, and 56% of coil. The tertiary structure analysis suggested that the Z-score and the Favored region of the HP13138PB vaccine were -4.47 88.22%, respectively. Furthermore, the binding energies of the HP13138PB to toll-like receptor 2 (TLR2) was -1224.7 kcal/mol. The immunoinformatics and real-world experiments showed that the HP13138PB vaccine could induce an innate and adaptive immune response characterized by significantly higher levels of cytokines such as interferon-gamma (IFN-γ), tumor necrosis factor-α (TNF-α), interleukin-4 (IL-4), and IL-10.

**Conclusion:**

The HP13138PB is a potential vaccine candidate to prevent TB, and this study preliminarily evaluated the ability of the HP13138PB to generate an immune response, providing a precursor target for developing TB vaccines.

## 1 Introduction

Tuberculosis (TB) is an infectious disease caused by infection with *Mycobacterium tuberculosis* (MTB), which is transmitted through the respiratory tract. MTB is an intracellular parasite that causes long-term infection mainly by attacking macrophages and inhibiting their apoptosis (1). In 2022, the report released by the World Health Organization (WHO) showed that there were 10.4 million new TB cases and 1.4 million deaths worldwide in 2021 (2). Since the 1990s, the WHO has developed a series of plans to stop TB from achieving the great goal of ending TB. However, since the outbreak of coronavirus disease 2019 (COVID-19), the number of case notifications of people newly diagnosed with TB has a partial recovery in 2021, to 6.4 million (2). These data suggested that TB is the second leading cause of death from a single infectious agent after COVID-19 (3).

Vaccination is the most effective way to prevent and control TB. The Bacillus Calmette-Guérin (BCG), the only licensed TB vaccine, had an excellent protective effect against miliary pulmonary tuberculosis and tuberculous meningitis in children (4, 5). However, its defensive efficiency for adult TB is very poor (0%-80%), and the protection period of BCG only maintains 10~20 years (6, 7). Candidate TB vaccines evaluated in clinical trials can be divided into four types: inactivated vaccines, live attenuated TB vaccines, subunit TB vaccines, and viral vector-based TB vaccines (8, 9). At present, the highly anticipated subunit vaccine M72/AS01E has completed phase 2 clinical trials. However, in 2019, the New England Journal of Medicine released the final data of the M72/AS01E vaccine in a phase 2b clinical trial, which included a total of 3500 adults aged 10-50 years, indicating that the overall vaccine effectiveness of M72/AS01E at 36 months after three years of follow-up was 49.7% (95% CI 2.1-74.2), below the WHO threshold of 50% protective efficacy (10). Therefore, the development of new TB vaccines is even more urgent.

With the rapid development of bioinformatics and immunoinformatics, the peptide-based vaccine has become one of the most attractive vaccine development strategies. The peptides identified from MTB antigens can be accurately characterized as chemical entities (similar to classic drugs) by a low-cost production technology (11). In addition, peptides are chemically defined compounds with good stability. The excellent properties of peptides have laid the advantage of easy transportation and preservation of peptide vaccines. Moreover, due to the absence of redundant elements, they can overcome some disadvantages observed in traditional vaccines, such as allergies and autoimmune reactions (11–13). As an interdisciplinary discipline based on informatics and modern immunology, the emergence of immunoinformatics has led to a change in vaccine research and development mode, which promoted the pace of research in the field of new TB vaccines (14, 15). Using bioinformatics tools, researchers can quickly and accurately deal with a large amount of data generated in the process of immune research, which greatly shortens the time for vaccine development (16, 17).

In this study, a new peptide-based TB vaccine was designed by using immunoinformatics technologies, and the antigenicity, immunogenicity, physicochemical parameters, secondary structure, tertiary structure, and immune stimulation of this vaccine were predicted and analyzed by immunoinformatics tools. Furthermore, the consistency of the immunoinformatics and real-world experiments results were investigated *via* enzyme-linked immunospot assay (ELISPOT) and Th1/Th2/Th17 cytokines detection experiments. This study will provide a new candidate for TB vaccine development.

## 2 Materials and methods

### 2.1 Bioinformatics prediction

#### 2.1.1 Selection of MTB antigens

Anat Zvi et al. screened 189 potential TB vaccine candidates from 3989 open reading frames of the whole genome of MTB through literature search and bioinformatics methods (18). Thirty-four of these antigens have been identified as potential TB vaccine candidates in previous studies. Among the 34 antigens, at least 5 antigens have been evaluated in clinical trials, such as Ag85A (Rv3804c), Ag85B (Rv1886c), ESAT-6 (Rv3875), MTB72F (Rv0125) and Rv1196. In addition, ten antigens have been used in animal models for protective research. The remaining 19 antigens were also shown to elicit strong immune responses (18). Therefore, five antigens assessed in clinical trials and 12 antigens used in the preclinical research stage were selected to predict and screen epitopes. These 17 candidate antigens were Ag85A, Ag85B, ESAT6, EspA, Mpt63, MTB32A, PPE18, RpfB, TB10.4, CFP10, MPT51, MPT64, MTB8.4, PPE44, PPE68, RpfA, and RpfB (**SupplementaryTable S1**).

#### 2.1.2 Selection of T cell epitopes

The Immune Epitope Database (IEDB) is a free database funded by the National Institutes of Allergy and Infectious Diseases (NIAID). It contains experimental data on antibody and T cell epitopes in humans, other primates, and some animals in the context of infectious diseases/autoimmunity. Therefore, helper T lymphocytes (HTL) and cytotoxic T lymphocytes (CTL) epitopes were predicted using the MHC II server (http://tools.iedb.org/mhcii/) (19) and the MHC I server (http://tools.iedb.org/mhci/) (20) in the IEDB database, respectively. Parameter selection in the major histocompatibility complex (MHC) II server: MHC allele(s): select full HLA reference set; length: 12-18. Parameter selection in the MHC I server: MHC allele(s): Select HLA allele reference set, length: All length. Furthermore, the percentile rank is obtained by comparing the peptide with the mer in the SWISSPROT database—the lower the percentile rank score, the stronger the binding ability to MHC. Finally, epitopes with a percentile rank < 0.5 were selected to be the dominant ones.

Subsequently, the dominant HTL and CTL epitopes were predicted for antigenicity, allergenicity, and toxicity. VaxiJen v2.0 is an alignment-independent protective antigen prediction server that classifies antigens based solely on the physicochemical properties of proteins without resorting to sequence alignment (21). VaxiJen v2.0 (http://www.ddg-pharmfac.net/vaxijen/VaxiJen/VaxiJen.html) was used to predict the antigenicity of these epitopes. The threshold was set as 0.5, and epitopes with antigenicity scores>0.7 were selected. Allergen FP v.1.0 is mainly used to identify known allergens and non-allergens (22). AllerTOP v.2.0 is currently the most accurate method for allergenicity prediction, with an accuracy rate of 88.7% (23). Herein, AllerTOP v.2.0 (http://www.ddg-pharmfac.net/AllerTOP/) and Allergen FP v.1.0 (http://ddg-pharmfac.net/AllergenFP/) were used to predict cellular epitope allergenicity. Toxicity of T epitopes predicted by Toxin Pred (http://crdd.osdd.net/raghava/toxinpred/) (24). Toxin Pred uses a unique electronic detection method for predicting the toxicity of peptides/proteins. Besides, it can also be used to design the least toxic peptides and discover toxic protein regions. Finally, the IFN epitope server (http://crdd.osdd.net/raghava/ifnepitope/index.php) was used to predict the IFN-γ inducibility of HTL cell epitopes (25). This server utilizes machine learning techniques, motifs-based search, and a hybrid approach to predict epitopes capable of inducing IFN production and activating CD4^+^ T lymphocytes. Moreover, the Class I Immunogenicity server (http://tools.iedb.org/immunogenicity/) was used to analyze the immunogenicity of CTL cell epitopes and screened epitopes with an immune score > 0 (26). Our previous studies have reported that the recognition between T cells and antigen present cells depend on peptides rather than the full-length protein, suggesting that peptide with a higher score might induce a stronger immune response (27, 28).

#### 2.1.3 Selection of linear B cell epitopes

The ABC pred Server (https://webs.iiitd.edu.in/raghava/abcpred/) was used to predict linear B cell epitopes (29). The parameter setting is as follows: length=20 amino acid residues and threshold=0.51. The server ranks predicted B-cell epitopes according to the scores obtained by the trained recurrent neural network. The higher the epitope score, the stronger the immune response that may be induced.

#### 2.1.4 Population coverage and construction of a peptide-based vaccine

Based on the HTL, CTL, and B cell epitopes predicted and screened by using the above bioinformatics tools: (1) the HTL epitopes with the highest adjusted rank, antigenicity, and IFN-γ scores, no toxicity, and no sensitization, (2) the CTL epitopes with the highest adjusted rank, immunogenicity and antigenicity scores, no toxicity, and no sensitization, (3) the B cell epitopes with the highest scores were finally identified as the candidate immunodominant epitopes for constructing the peptides-based vaccine.

The population coverage of the selected immunodominant HTL and CTL epitopes was performed using the Population Coverage tool in the IEDB database (http://tools.iedb.org/population/). HLA allele genotypic frequencies used in the IEDB database were obtained from the Allele Frequency database (http://www.allelefrequencies.net/). This database provides allele frequencies for 115 countries and 21 ethnicities grouped into 16 geographical areas. The novel peptide-based vaccine consists of four components and is named HP13138PB. First, the selected epitopes were connected by amino acid linkers (GPGPG, AAY, KK). Then, antimicrobial peptide human β-defensin 3 (HBD-3, GIINTLQKYYCRVRGGRCAVLSCLPKEEQIGKCSTRGRKCCRRKK) (30) and TLR2 agonist phenol-soluble modulin α4 (PSMα4, MAIVGTIIKIIKAIIDIFAK) (31) were added to the beginning and end of the amino acid sequence to enhance vaccine’s immunogenicity. Furthermore, the pan HLA DR-binding epitope (PADRE) (AGLFQRHGEGTKATVGEPV) was added following the carboxyl-terminal adjuvant (32). Finally, a 6-His tag was added at the end of the amino acid sequence.

Additionally, the antigenicity, allergenicity, immunogenicity, and toxicity of the constructed peptide-based vaccine were predicted and analyzed with VaxiJen v2.0 (21)、ANTIGENpro (33)、AllerTOP v.2.0 (23), Allergen FP v.1.0 (22), IEDB Immunogenicity server (26), and Toxin Pred server (24), respectively.

#### 2.1.5 Physicochemical properties and secondary structure of the peptide-based vaccine

The Expasy Protparam server (https://web.expasy.org/protparam/) was used to predict the physicochemical parameters of the peptide-based vaccine. It can predict the vaccine’s physicochemical properties, such as molecular weight, theoretical pI, amino acid composition, atomic composition, extinction coefficient, estimated half-life, instability index, aliphatic index, and grand average of hydropathicity (GRAVY) (34). The Protein–Sol server (https://protein-sol.manchester.ac.uk/) was used to predict the solubility of peptide-based vaccines (35). The single amino acid sequence obtained by the Protein-Sol server was compared with the data in the database. The solubility value > 0.45 means that the protein has better solubility.

The PSIPRED server (http://bioinf.cs.ucl.ac.uk/psipred/) was used to generate the secondary structure of the peptide-based vaccines. It can effectively identify the transmembrane topology, transmembrane helix, fold and domain recognition, etc. (36). The RaptorX Property (http://raptorx.uchicago.edu/StructurePropertyPred/predict/) was used to predict secondary structure characteristics of peptide-based vaccines (37). The server uses an evolving machine learning model called Deep Convolutional Neural Fields (Deep CNF) to continuously calculate secondary structure (SS), disorder regions (DISO), and solvent accessibility (ACC). The secondary structure includes alpha-helix, beta-sheet, and coil. The solvent accessibility is divided into three states, buried for less than 10%, exposed for larger than 40%, and medium for between 10% and 40%. Order/disorder prediction is based on the cutoff value at 0.25.

#### 2.1.6 Tertiary structure prediction, optimization, and validation of peptide-based vaccine

The I-TASSER server can automatically find templates from protein databases for the structure prediction of molecules through the multi-threaded method LOMETS (38). Therefore, the I-TASSER server (https://zhanggroup.org//I-TASSER/) was used to predict the three-dimensional spatial structure of the vaccine. Then, the tertiary structure of the peptide-based vaccine was optimized using the GalaxyRefine web server (https://galaxy.seoklab.org/cgi-bin/submit.cgi?type=REFINE) to refine the side chains and perform side-chain re-packing following a previous study (39).

The ProSA-web server (https://prosa.services.came.sbg.ac.at/prosa.php) and the ERRAT server (https://saves.mbi.ucla.edu/) were used to verify the structure of the peptide-based vaccine to show possible errors (40, 41). The ProSA-web server uses Z-score to deliver the potential errors in the protein structure, and Z-score > 0 indicates that an error or unstable part has been found in the protein model. In addition, the SWISS-MODEL server (https://swissmodel.expasy.org/assess) was used to draw Ramachandran diagrams of peptide-based vaccines (42). A Ramachandran plot is a way to visualize energetically favored regions for backbone dihedral angles against amino acid residues in protein structure. Histograms with a binning of 4 degrees were used to count Φ (Phi; C-N-CA-C)/Ψ (Psi; N-CA-C-N) occurrences for all displayed categories.

#### 2.1.7 Conformational B cell epitopes

Conformational epitopes play a significant role in stimulating immune responses. The constructed protein folds to form conformational B-cell epitopes, of which more than 90% of B-cell epitopes are determined to be discontinuous. Compared to other structure-based methods for predicting epitopes, ElliPro achieves the highest level and provides an area under the receiver operator characteristic curve (AUC) value of (0.732) as the best calculation for any protein. Therefore, the conformational B cell epitopes were predicted using the ElliPro server (http://tools.iedb.org/ellipro/) (43).

#### 2.1.8 Molecular docking of peptide-based vaccine with related antigen-recognition receptors

Molecular docking is calculated by computer to obtain stable receptor-ligand complexes and predict the binding affinity between them according to the scoring function. Therefore, we assessed the interaction between the peptide-based vaccine and Toll-like receptors (TLRs). The protein data bank (PDB) structure file for TLR2 (PDB ID: 6NIG) was obtained from the NCBI Molecular Modeling Database (MMDB) (https://www.ncbi.nlm.nih.gov/structure/). Subsequently, molecular docking was performed using the ClusPro2.0 server (https://cluspro.bu.edu/home.php) to verify the interaction between the TLRs and peptide-based vaccine (44). The server analyzed the molecular docking of peptide-based vaccine with TLR through the following three steps: (1) rigid body docking by sampling billions of conformations; (2) root-mean-standard deviation (RMSD) based clustering of the 1000 lowest energy structures generated to find the largest clusters; (3) removal of steric clashes using energy minimization. Finally, Hydrogen bonds and hydrophobic interactions were evaluated with the LigPlot^+^ program (45).

#### 2.1.9 Molecular dynamic simulation

Molecular dynamics simulations are essential for determining stability between receptor-ligand complexes. Simulation predictions can enhance understanding of the microstructure of the interaction between peptide-based vaccines and Toll-like receptors (46). Gromacs v5.1.515 (47) was used to determine the structural properties and interactions between ligands (vaccine) and receptors (TLR2). All molecular dynamics simulations were performed under the AMBER99 force field. At the same time, energy minimization was performed before the simulation to ensure the correct geometry of the system, and the steepest descent algorithm method was used to avoid spatial conflicts. During the equilibration phase (100 ps), the temperature increased to 300 K and pressure up to 1 bar.

#### 2.1.10 Immune simulations

The C-ImmSim server (https://150.146.2.1/C-IMMSIM/index.php) was used to predict immune simulation (48). This server can evaluate the immune response of the B and T lymphocytes (including Th1 and Th2 lymphocytes) under a simulated vaccine injection state. Then, the C-ImmSim server parameters were set as Random Seed=12345, Simulation Volume=10, Simulation Steps=1000, and the host alleles HLA-A0101, A0201, B0702, B0801, DRB10101, and DRB1501 were selected. Finally, we predicted the cellular immune response and cytokine levels induced by the peptide-based vaccine with three vaccine injections.

### 2.2 Experimental verification

#### 2.2.1 Ethics and experimental subjects

Twenty-one healthy controls, 24 individuals with latent TB infection (LTBI), and 18 patients with active tuberculosis (ATB) were recruited. The recruitment was conducted from April 2022 to September 2022 at the 8th Medical Center of the PLA General Hospital. The TB diagnostic criteria (WS288-2017) formulated by the National Health and Family Planning Commission (NHFPC) were used to determine patients with ATB and individuals with LTBI. The clinical experiments on blood samples collected from participants were approved by the Medical Ethics Committee of the Eighth Medical Center of the PLA General Hospital (Approval No: 309202204080808). The informed consent form was obtained from each participant.

#### 2.2.2 Plasmid construction and expression of vaccine

The nucleotide of the vaccine was synthesized by the Wuhan Dangang Biotechnology Co., Ltd and inserted into the pET28a(+) plasmid through the restriction sites BamHI and XhoI to transform *Escherichia coli* (*E. coli*) BL21 *in vitro*. The expression and purification of the vaccine were executed by the C-terminal 6-his tag according to our previous study (49). In brief, the synthesized plasmids were transformed into *E. coli* BL21(DE3) competent cells and plated on LB solid plates (100 µg/ml kanamycin) and cultured overnight at 37°C. Then, single colonies were picked and inoculated in 5ml of liquid LB medium (100 µg/ml kanamycin) at 37°C, 220 rpm, and incubated overnight. One milliliter of the first-passage strains was added into 100ml liquid LB medium (100 µg/ml kanamycin) and incubated at 37°C for 4 to 6h at 220 rpm. The second-generation strains were inoculated into 1L liquid LB medium (15 µg/ml kanamycin) at 1% ratio and cultured at 37°C at 220rpm until the OD value of the bacterial solution was 0.6-0.8. The vaccine protein expression was induced with IPTG at a final concentration of 0.1mM overnight at 16°C and 220 rpm. The bacteria were collected by centrifugation at 8000rpm for 10min, resuspended by adding the breaking solution (W/V=1:15), and then crushed twice by a high-pressure homogenizer at 1000 bar. After fragmentation, the cells were centrifuged at 8000 rpm for 45 min at 4°C, and the supernatant was collected. Finally, the vaccine protein was purified with the C-terminal 6-his tag by using Ni-affinity chromatography.

#### 2.2.3 ELISPOT experiment

The blood samples (5ml) were collected from healthy controls (HCs), LTBI individuals, and ATB patients to separate peripheral blood mononuclear cells (PBMCs). A part of the isolated PBMCs was added into a 96-well ELISPOT culture plate (2.5×10^5^ cells/well) and stimulated with 50μl of HP13138PB (100μg/ml). The culture plate was incubated in a CO_2_ incubator at 37°C. Twenty-four hours later, the spot number of interferon-gamma positive (IFN-γ)^+^ T cells was detected by using Human IFN-γ ELISpot^PRO^ Kit (Cat. No. 3420-2APW-10, Mabtech AB, Nacka Strand, Sweden) according to the manufacturer’s instructions.

#### 2.2.4 Detection of Th1/Th2/Th17 cytokines

The remaining PBMCs isolated from participants were added to a 96-well cell culture plate (2.5×10^5^ cells/well) (Mabtech AB, Nacka Strand, Sweden). Then, the PBMCs were stimulated with 50μl of HP13138PB (100μg/ml) and incubated in a CO_2_ incubator at 37°C for 48 hours. The PBMCs and cell culture medium mixture was transferred to a new tube and centrifuged at 500 g for 10 min. Finally, the supernatant was gently transferred to another new tube, and levels of interleukin-2 (IL-2), IL-4, IL-6, IL-10, IFN-γ, tumor necrosis factor-α (TNF-α), and IL-17A were measured with a human Th1/Th2/Th17 cytokine kit (Cat 560485) following our previous studies (28, 49, 50).

#### 2.2.5 Statistical analysis

The data obtained from the ELISPOT assay and cytokines detection were conducted using GraphPad Prism 9.4.1 software (San Diego, CA, USA). The results of the ELISPOT assay were analyzed using either an unpaired t-test or a nonparametric test (Mann Whitney test) based on normality. The cytokines detection results were analyzed with the Ordinary one-way ANOVA test or Kruskal-Wallis nonparametric test according to the data normality and homogeneity of variances. The data were presented as mean ± standard error of the mean (SEM), and *P*<0.05 was considered a significant difference.

## 3 Results

### 3.1 Selection of immunodominant HTL, CTL, and B cell epitopes

Seventeen MTB protective antigens were selected to predict immunodominant HTL, CTL and B cell epitopes with good antigenicity, immunogenicity, non-allergenicity and non-toxicity, including Ag85A, Ag85B, ESAT6, EspA, Mpt63, MTB32A, PPE18, RpfB, TB10.4, CFP10, MPT51, MPT64, MTB8.4, PPE44, PPE68, RpfA, and RpfB. As a result, 203 HTL epitopes were screened from these antigens, and 31 representative HTL epitopes (marked with blue background color in **Supplementary Table S2**) were finally selected for further exploration (**Supplementary Table S2**). A total of 28 CTL epitopes were identified from these antigens (**Supplementary Table S3**). In addition, 46 B cell epitopes with a prediction score >0.85 were determined using ABCpred Prediction Server (**Supplementary Table S4**).

### 3.2 Construction of peptide-based vaccine HP13138PB

Thirteen HTL epitopes (**Table 1**), 13 CTL epitopes (**Table 2**), and 8 B cell epitopes (**Table 3**) with the highest rank, antigenicity, immunogenicity, no allergenicity, and no toxicity were selected to construct a peptide-based vaccine termed HP13138PB. The population coverage of the HTL and CTL immunodominant epitopes of the HP13138PB was determined by the population coverage tool in the IEDB database (**Table 4**). Our results showed that the population coverage rates of CTL epitopes (Class I) of the HP13138PB vaccine in Central Africa, Central America, East Africa, East Asia, Europe, North Africa, North America, Northeast class in Asia, Oceania, South America, South Asia, Southeast Asia, Southwest Asia, West Africa, West Indies, and the world were 68.55%, 2.78%, 78.26%, 82.31%, 96.91%, 86.09%, 92.37%, 83.48%, 68.69%, 80.54%, 85.36%, 70.17%, 83.21%, 81.57%, 90.04%, and 92.07%, respectively. Similarly, the population coverage rates of HTL epitopes (Class II) of the HP13138PB vaccine in Central Africa, Central America, East Africa, East Asia, Europe, North Africa, North America, Northeast class in Asia, Oceania, South America, South Asia, Southeast Asia, Southwest Asia, West Africa, West Indies, and the world were 75.27%, 98.55%, 83.73%, 67.46%, 69.62%, 73.75%, 86.59%, 90.66%, 94.22%, 91.72%, 66.72%, 83.45%, 68.85%, 86.21%, 72.98%, and 73.60%, respectively.

**Table 1:** The dominant HTL epitopes selected to construct the vaccine.

Antigen	Allele	Start	End	Length	Peptide	Adjusted rank	Antigenicity Scores	IFN-γ scores	AllerTOP v.2.0^*^	Aller FP^*^	Toxin Pred
Ag85A	HLA-DQA1*06:01/DQB1*03:03	160	175	16	PTGSAVVGLSMAASSA	0.47	0.7318	0.91052778	2	2	Non-Toxin
Ag85B	HLA-DQA1*05:01/DQB1*03:01	26	39	14	PGLVGLAGGAATAG	0.47	0.8884	0.56955554	2	2	Non-Toxin
CFP10	HLA-DQA1*03:01/DQB1*06:01	74	86	13	TNIRQAGVQYSRA	0.31	0.7348	0.61651125	2	2	Non-Toxin
MPT51	HLA-DQA1*03:01/DQB1*06:01	145	158	14	AQGGYGAMALAAFH	0.33	0.8205	0.024282193	2	2	Non-Toxin
MPT64	HLA-DRB3*02:02	43	59	17	IQMSDPAYNINISLPSY	0.46	1.1439	0.7822075	2	2	Non-Toxin
MTB8.4	HLA-DQA1*06:01/DQB1*03:03	13	28	16	GAVAMSLTVGAGVASA	0.45	0.9583	0.40174423	2	2	Non-Toxin
MTB32A	HLA-DQA1*05:01/DQB1*03:01	137	152	16	GLPSAAIGGGVAVGEP	0.18	0.8414	0.21165345	2	2	Non-Toxin
PPE18	HLA-DQA1*01:02/DQB1*06:02	81	98	18	AGQAELTAAQVRVAAAAY	0.06	1.0066	0.99194898	2	2	Non-Toxin
PPE44	HLA-DQA1*06:01/DQB1*03:03	61	78	18	ASLSMAAAVQPYLVWLTC	0.38	0.7694	0.56423563	2	2	Non-Toxin
	HLA-DQA1*03:01/DQB1*06:01	147	164	18	DASAMYGYAAASAVAARL	0.03	0.7116	0.68087264	2	2	Non-Toxin
	HLA-DQA1*06:01/DQB1*03:03	147	164	18		0.06	0.7116	0.68087264	2	2	Non-Toxin
	HLA-DQA1*05:01/DQB1*03:01	147	164	18		0.12	0.7116	0.68087264	2	2	Non-Toxin
	HLA-DRB1*16:02	147	164	18		0.38	0.7116	0.68087264	2	2	Non-Toxin
PPE68	HLA-DQA1*03:01/DQB1*06:01	304	319	16	VAPSVMPAAAAGSSAT	0.31	1.0173	0.23735279	2	2	Non-Toxin
RpfA	HLA-DQA1*06:01/DQB1*03:03	27	40	14	GGGIAMAAQATAAT	0.16	0.922	0.13912009	2	2	Non-Toxin
	HLA-DQA1*03:01/DQB1*06:01	27	40	14		0.18	0.922	0.13912009	2	2	Non-Toxin
RpfB	HLA-DQA1*05:01/DQB1*03:01	9	24	16	LLLVLAFAGGYAVAAC	0.12	0.7437	0.26811457	2	2	Non-Toxin

^*^, AllerTOP v.2.0 and Allergen FP v.1.0 were used to predict allergenicity. 1 stands for allergenicity and 2 stands for non- allergenicity.

**Table 2:** The dominant CTL epitopes selected to construct the vaccine.

Protein	Peptide	Allele	Length	Immunogenicity score	Antigenicity score	AllerTOP v.2.0^*^	Allergen FP v.1.0^*^	Toxicity
Ag85A	GLPVEYLQV	HLA-A*02:01, HLA-A*02:03, HLA-A*02:06	9	0.05492	0.7069	2	2	Non-Toxin
Ag85B	NAAGGHNAVF	HLA-A*68:02, HLA-B*35:01, HLA-B*51:01, HLA-B*15:01	10	0.16235	1.4758	2	2	Non-Toxin
CFP10	ELDEISTNI	HLA-A*02:01, HLA-A*68:02, HLA-A*02:06, HLA-A*02:03	9	0.1108	0.7491	2	2	Non-Toxin
MPT51	AMSGDIVGA	HLA-A*02:03, HLA-A*02:01, HLA-A*02:06, HLA-B*15:01	9	0.18192	1.0252	2	2	Non-Toxin
MPT64	GGTHPTTTY	HLA-A*30:02, HLA-B*15:01, HLA-A*01:01	9	0.12633	1.7475	2	2	Non-Toxin
MTB32A	RAVPGRVVAL	HLA-A*02:06, HLA-B*07:02, HLA-B*08:01, HLA-A*02:03, HLA-A*02:01	10	0.1822	1.1311	2	2	Non-Toxin
PPE18	ATATATATL	HLA-A*68:02, HLA-A*02:06, HLA-A*32:01, HLA-B*07:02	9	0.1821	1.0012	2	2	Non-Toxin
PPE44	SAIAATEAR	HLA-A*68:01, HLA-A*31:01, HLA-A*33:01, HLA-A*11:01	9	0.26457	0.8366	2	2	Non-Toxin
PPE68	QAVELTARL	HLA-A*68:02, HLA-B*51:01, HLA-B*35:01	9	0.20315	0.7764	2	2	Non-Toxin
RpfA	VLGGGGIAM	HLA-B*15:01	9	0.24518	0.9451	2	2	Non-Toxin
RpfA	LSNATPREV	HLA-B*51:01	9	0.16681	0.7995	2	2	Non-Toxin
RpfB	QVTRNRIKK	HLA-A*11:01, HLA-A*68:01, HLA-A*30:01, HLA-A*03:01	9	0.09342	0.7561	2	2	Non-Toxin
RpfE	ASREEQIRV	HLA-A*30:01	9	0.24857	1.4212	2	2	Non-Toxin

^*^, AllerTOP v.2.0 and Allergen FP v.1.0 were used to predict allergenicity. 1 stands for allergenicity and 2 stands for non- allergenicity.

**Table 3:** The dominant B cell epitopes selected to construct the vaccine.

Protein	Sequence	Start position	End	Length	Score
Ag85B	AVYLLDGLRAQDDYNGWDIN	75	94	20	0.89
ESAT6	GVQQKWDATATELNNALQNL	53	72	20	0.89
EspA	TKKYSEGAAAGTEDAERAPV	357	376	20	0.89
Mpt63	TAVIPGYPVAGQVWEATATV	63	82	20	0.89
MTB32A	DSLTGAEETLNGLIQFDAAI	184	203	20	0.87
PPE18	VRAMSSLGSSLGSSGLGGGV	286	305	20	0.89
RpfB	LATREEQIAVAEVTRLRQGW	330	349	20	0.88
TB10.4	AYHAMSSTHEANTMAMMARD	68	87	20	0.86

**Table 4:** Population coverage calculation results of the vaccine.

population/area	Class I			Class II		
	Coverage ^a^	Average hit ^b^	pc90 ^c^	Coverage ^a^	Average hit ^b^	pc90 ^c^
Central Africa	68.55%	2.08	0.32	75.27%	1.64	0.4
Central America	2.78%	0.04	0.1	98.55%	5.5	3.28
East Africa	78.26%	2.61	0.46	83.73%	2.18	0.61
East Asia	82.31%	3.88	0.57	67.46%	2.78	0.31
Europe	96.91%	4.53	1.69	69.62%	2.35	0.33
North Africa	86.09%	3.04	0.72	73.75%	2.4	0.38
North America	92.37%	4.05	1.22	86.59%	3.73	0.75
Northeast Asia	83.48%	3.41	0.61	90.66%	4.37	1.05
Oceania	68.69%	1.9	0.32	94.22%	4.63	1.36
South America	80.54%	2.35	0.51	91.72%	4.59	1.32
South Asia	85.36%	2.82	0.68	66.72%	2.56	0.3
Southeast Asia	70.17%	2.6	0.34	83.45%	3.61	0.6
Southwest Asia	83.21%	2.85	0.6	68.85%	2.5	0.32
West Africa	81.57%	2.75	0.54	86.21%	2.34	0.73
West Indies	90.04%	3.49	1	72.98%	2.2	0.37
World	92.07%	3.88	1.16	73.60%	2.8	0.38
Average	77.39	2.86	0.66	80.21	3.14	0.78
Standard deviation	20.34	1.01	0.39	10.07	1.08	0.73

a Projected population coverage.

b Average number of epitope hits/HLA combinations recognized by the population.

c Minimum number of epitope hits/HLA combinations recognized by 90% of the population.

**Figure 1:**
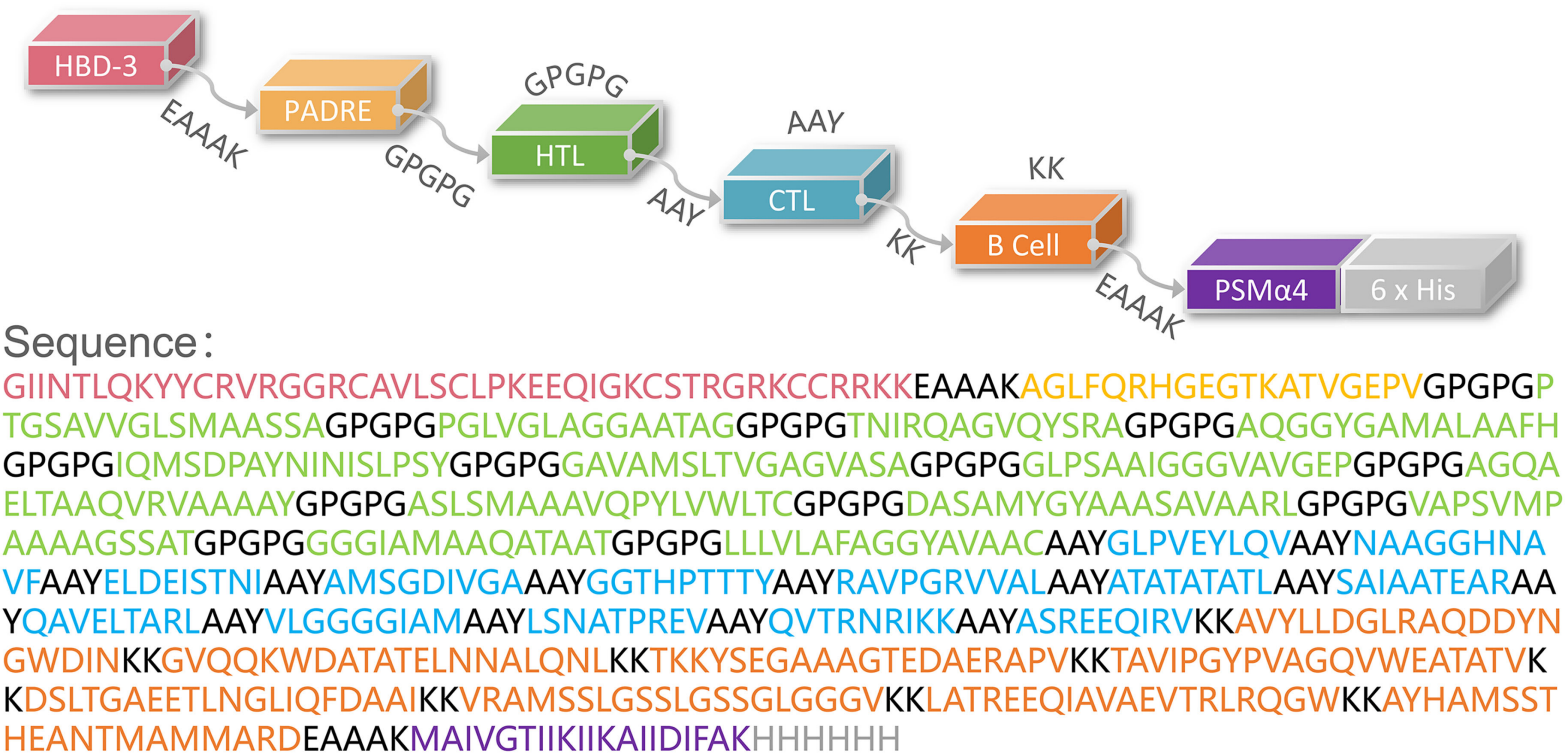
Schematic representation of the HP13138PB vaccine. The amino acid sequence of the epitopes, adjuvants, Pan DR reactive epitope (PADRE), and linkers have been shown in different colors.

Then, these HTL, CTL, and B cell epitopes were connected with the GPGPG, AAY, and KK linkers, respectively. Finally, the HBD-3 and PADRE were added to the amino terminus, and the TLR2 agonist PSMα4 and a 6-His tag were added to the carboxyl terminus (**Figure 1**).

### 3.3 Physicochemical properties and secondary structure of the HP13138PB vaccine

**Figure 2:**
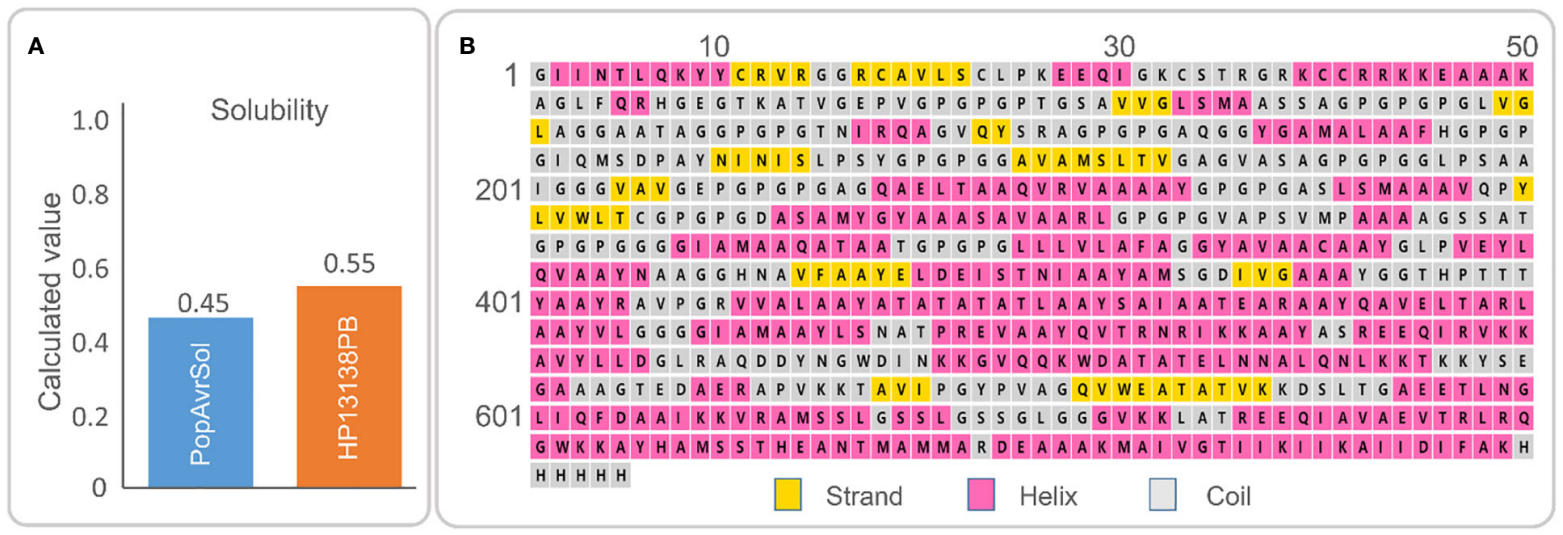
**(A)** The molecular solubility of the HP13138PB vaccine was predicted by the Protein-Sol server. **(B)** The secondary structure of the HP13138PB vaccine.

The HP13138PB vaccine was composed of 705 amino acids. According to Expasy Protparam server analysis, its molecular weight was 70245.98 Da, theoretical pI was 9.41, and the estimated half-life was 30 hours (mammalian reticulocytes, *in vitro*), >20 hours (yeast, *in vivo*), or >10 hours (*Escherichia coli*, *in vivo*). Furthermore, we also found that the instability index, aliphatic index, and Grand average of hydropathicity (GRAVY) were 33.20, 79.32, and 0.04, respectively (**Table 5**). In addition, the solubility of the HP13138PB vaccine predicted by Protein–Sol Server was 0.55, which was higher than the average threshold of 0.45 (**Figure 2A**), indicating that the HP13138PB vaccine has good solubility.

**Table 5:** Physicochemical property of HP13138PB predicted by the Expasy Protparam.

Number of amino acids	Weight	Theoretical pI	Estimated half-life:	Instability index:	Aliphatic index	GRAVY
705 aa	70245.98	9.41	30 hours (mammalian reticulocytes, *in vitro*). >20 hours (yeast, *in vivo*). >10 hours (Escherichia coli, *in vivo*).	33.20	79.32	0.04

The secondary structure of HP13138PB was shown in **Figure 2B**. The previous study indicated that the α- Helix and natural unfolded protein regions are important “structural antigens” types, which are beneficial for naturally induced antibody recognition after infection (51). Our results showed that the HP13138PB vaccine contained 31% α-helix, 11% β-strand, and 56% coil. Furthermore, 55% of amino-acid residues were expected to be exposed, 12% medium exposed, and 31% buried in support of solvent accessibility.

### 3.4 Tertiary structure prediction and validation of the HP13138PB vaccine

**Figure 3:**
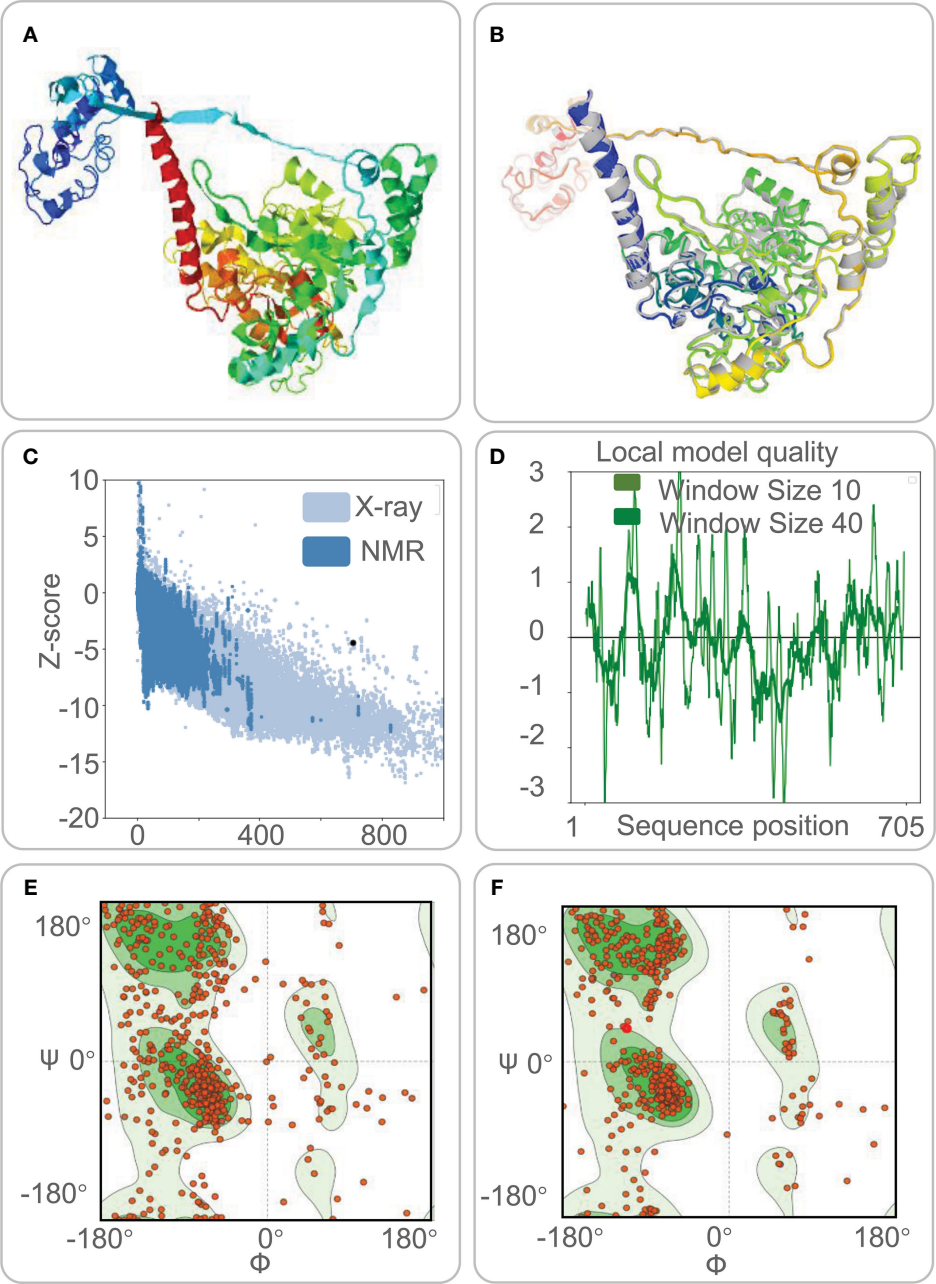
The three-dimensional (3-D) model, Z-score, and Ramachandran diagram of the HP13138PB vaccine. **(A)** The model of the HP13138PB vaccine was predicted by the I-TASSER server. **(B)** The Galaxy Refine web server indicated a model of the HP13138PB vaccine. The colored part repackaged the amino acid side chain and the overall structural relaxation that minimizes energy. **(C)** The Z-score of the HP13138PB vaccine was -4.47 determined by the ProSA-web server. The light blue and dark blue represent the Z-score of all proteins in the PDB experimentally solved by X-Ray and NMR (nuclear magnetic resonance) spectroscopy, respectively. **(D)** The amino acid sequence position of the HP13138PB vaccine showed local model quality by the ProSA-web server. **(E)** The Ramachandran diagram before optimization. The two green areas represent the favored region and the outlier region. The white area is the rotamer region. The favored, outlier, and rotamer regions of the HP13138PB vaccine were 56.97%, 14.09%, and 14.25%. **(F)** The Ramachandran diagram after optimization. The favored, outlier, and rotamer regions of the HP13138PB vaccine were 88.22%, 4.13%, and 0.88%.

Five 3-D models were predicted by the I-TASSER server, and their C-scores were −1.65, −2.82, −4.30, −3.25, and −2.38, respectively. In general, the C-score was between -2 and 5, and the larger the value, the higher the accuracy of the model. Herein, we choose a model with a C-score of -1.65, with a TM score of 0.51 ± 0.15, and the expected root-mean-square deviation (RMSD) was 12.1 ± 4.4A (**Figure 3A**). Subsequently, we used the GalaxyRefine web server to refine the loops and minimize energy in the model to improve the consistency of the modeled proteins. The GDT-HA represents the accuracy of the backbone structure in which the model was built. The MolProbity scores include clash score, poor rotamers, and Rama favored. It represents various interactions at the atomic level of the model, and the smaller the value, the better the quality of the model structure. Therefore, higher GDT-HA values and lower MolProbity values indicate better model quality. As a result, model 4 was selected as the final three-dimensional (3-D) model of the HP13138PB vaccine (**Figure 3B**) from the five optimization models (**Table 6**).

**Table 6:** The GalaxyRefine web server predicted the results of the HP13138PB molecular 3-D model.

Model	GDT-HA	RMSD	MolProbity	Clash score	Poor rotamers	Rama favored
Initial	1.0000	0.000	3.415	12.6	14.7	59.5
MODEL 1	0.9209	0.494	2.647	26.3	1.1	81.9
MODEL 2	0.9216	0.488	2.796	25.9	1.8	81.9
MODEL 3	0.9177	0.490	2.871	26.4	2.2	82.4
MODEL 4	0.9177	0.498	2.594	25.1	0.7	82.2
MODEL5	0.9184	0.501	2.612	25.2	0.7	81.1

The ProSA-web server and ERRAT-web server were used to validate 3-D models of the HP13138PB vaccine. The ProSA-web server predictions show that the Z-score of the HP13138PB vaccine was -4.47 (**Figure 3C**), and the energy graph was shown in **Figure 3D**. However, the Z-score of the HP13138PB vaccine deviated from the Z-score range of most experimentally determined protein chains in the current PDB. The amino acid misfolding part in the protein folding structure corresponds to the positive value of the energy map. The result shows fewer wrong parts in the model, indicating that the overall structure is acceptable. The ERRAT-web server predicted that the Overall Quality Factor of the HP13138PB vaccine was 62.03%. In addition, the Ramachandran plot showed that the favored region in the HP13138PB vaccine rose from 59.67% to 88.22% after optimization, the outlier region from 14.09% to 4.13%, and the rotamer region from 14.25% to 0.88% (**Figures 3E, F**).

### 3.5 Conformational B−cell epitopes

**Figure 4:**
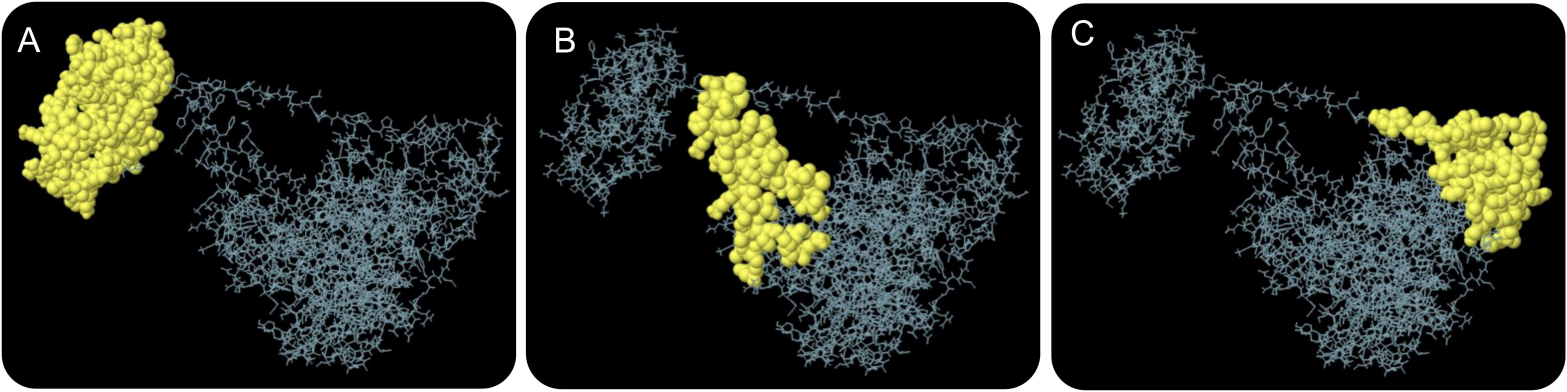
The conformational B-cell epitopes of the HP13138PB vaccine predicted by the ElliPro server. Three conformational B-cell epitopes **(A–C)** were predicted and identified in the amino acid sequence of HP13138PB vaccine, which are indicated by yellow balls and other amino acid residues in their amino acid sequence are indicated by gray lines.

The ElliPro server was used to predict conformational B-cell epitopes. Our results showed that a total of 267 residues were distributed on three conformational B-cell epitopes (**Figure 4** and **Table 7**).

**Table 7:** The conformational B cell epitope residues of HP13138PB predicted by the ElliPro server.

No	Residues	Number of residues	Score
1	A:G1, A:I2, A:I3, A:N4, A:T5, A:L6, A:Q7, A:K8, A:Y9, A:Y10, A:C11, A:R12, A:V13, A:R14, A:G15, A:G16, A:R17, A:C18, A:A19, A:V20, A:L21, A:S22, A:C23, A:L24, A:P25, A:K26, A:E27, A:E28, A:Q29, A:I30, A:G31, A:K32, A:C33, A:S34, A:T35, A:R36, A:G37, A:R38, A:K39, A:C40, A:C41, A:R42, A:R43, A:K44, A:K45, A:E46, A:A47, A:A48, A:A49, A:K50, A:A51, A:G52, A:L53, A:F54, A:Q55, A:R56, A:G58, A:E59, A:G60, A:T61, A:K62, A:A63, A:T64, A:V65, A:G66, A:E67, A:P68, A:V69, A:G70, A:P71, A:G72, A:P73, A:G74, A:P75, A:T76, A:G77, A:S78, A:A79, A:V80, A:V81, A:G82, A:L83, A:S84, A:M85, A:A86, A:A87, A:S88, A:S89, A:A90, A:G91, A:P92, A:G93, A:P94, A:G95, A:P96, A:G97, A:L98, A:V99, A:G100, A:L101, A:A102, A:G103, A:G104, A:A105, A:A106, A:T107, A:A108, A:G109, A:G110, A:P111, A:G112, A:P113, A:G114, A:T115, A:N116, A:I117, A:R118, A:Q119, A:A120, A:G121, A:V122, A:Q123, A:Y124, A:S125, A:R126, A:A127, A:G128, A:P129, A:G130, A:P131, A:G132, A:A133, A:Q134, A:G135, A:G136, A:Y137, A:G138, A:A139, A:M140, A:A141, A:L142, A:A143, A:A144, A:F145, A:H146, A:G147, A:P148, A:G149, A:P150, A:G151, A:I152, A:Q153	152	0.814
2	A:G303, A:P304, A:G305, A:G306, A:G307, A:G308, A:I309, A:A310, A:M311, A:A312, A:A313, A:Q314, A:A315, A:T316, A:G628, A:G629, A:K631, A:K632, A:D674, A:A677, A:A678, A:A681, A:I682, A:G684, A:T685, A:I686, A:I687, A:K688, A:I689, A:I690, A:K691, A:A692, A:I693, A:I694, A:D695, A:I696, A:F697, A:A698, A:K699, A:H700, A:H701, A:H702, A:H703, A:H704, A:H705	45	0.724
3	A:Y168, A:G169, A:P170, A:G171, A:P172, A:G173, A:G174, A:A175, A:V176, A:A177, A:M178, A:S179, A:L180, A:T181, A:V182, A:G183, A:A184, A:G185, A:V186, A:A187, A:S188, A:A189, A:G190, A:P193, A:G194, A:S242, A:A244, A:A245, A:A246, A:V247, A:Q248, A:P249, A:Y250, A:L251, A:V252, A:W253, A:L254, A:T255, A:C256, A:G257, A:P258, A:G259, A:P260, A:G261, A:D262, A:A263, A:S264, A:A265, A:M266, A:Y267, A:G268, A:Y269, A:A270, A:A271, A:A272, A:S273, A:A274, A:V275, A:A276, A:A277, A:R278, A:L279, A:G280, A:P281, A:G282, A:P283, A:G284, A:A286, A:P287, A:S288	70	0.699
4	A:E369, A:K546, A:K547, A:S549, A:E550, A:G551, A:A552, A:A553, A:A554, A:G555, A:T556, A:E557, A:D558, A:A559, A:E560, A:R561, A:A562, A:P563, A:V564, A:K565, A:A606, A:A607, A:I608, A:K609, A:K610, A:V611, A:R612, A:M614, A:H657, A:A658, A:M659, A:S660	32	0.635
5	A:K485, A:A487, A:A488, A:Y489, A:A490, A:S491, A:R492, A:E493, A:E494, A:Q495, A:I496, A:R497, A:V498, A:K499, A:A501	15	0.602
6	A:G204, A:V205, A:A206, A:V207, A:G208, A:E209, A:P210, A:G211, A:P214, A:G215, A:A216, A:G217, A:Q218, A:A219, A:E220, A:L221, A:T222, A:A223, A:A224, A:Q225, A:V226, A:R227, A:V228, A:A229, A:A230, A:A336, A:V337, A:A338, A:A339, A:C340, A:A341, A:A342, A:E348, A:Y349, A:L350, A:Q351, A:V352, A:A353, A:A354, A:Y355, A:N356, A:A357, A:A358, A:G359, A:G360, A:H361, A:N362, A:A363, A:V364, A:F365, A:A366, A:A367, A:Y368, A:L370, A:D371, A:I373, A:S374, A:T375, A:L633, A:A634, A:T635, A:M668, A:M671, A:A672, A:R673	65	0.582

### 3.6 Molecular docking

**Figure 5:**
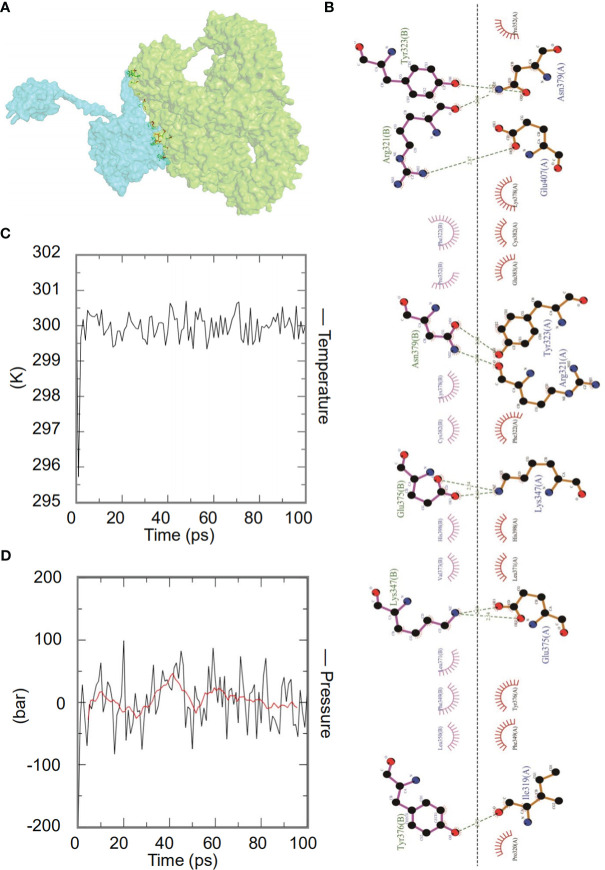
The interaction of the HP13138PB vaccine with toll-like receptor 2 (TLR2). **(A)** Diagram of HP13138PB docking with TLR2 molecule. Blue represents the HP13138PB vaccine, and green means the TLR2 receptor. **(B)** The interaction of the HP13138PB vaccine and the TLR2 was described in a 2D plot. The B chain represents the residence of the HP13138PB vaccine with hydrogen bonds. The A chain represents the residue of the TLR2 receptor with hydrogen bonds. The temperature plot and the pressure graph of HP13138PB-TLR2. **(C, D)** The temperature plot and the pressure graph of HP13138PB-TLR2 at 300K and 1 bar under the AMBER99 force field using Gromacs v5.1.515.

The ClusPro2.0 server was used for molecular docking between the HP13138PB vaccine and TLRs, generating 30 model complexes. We selected the model with the lowest binding energy between HP13138PB-TLR2 (**Figures 5A**). The combined energies of the model was -1224.7 kcal/mol. Subsequently, we used the LigPlot^+^ program to show hydrophobic interactions between the HP13138PB vaccine and TLR2. The results indicated that nine hydrogen bonds and nine hydrophobic interactions were observed between HP13138PB and TLR2 (**Figure 5B**).

### 3.7 Molecular dynamics simulation

Dynamics simulation prediction of the HP13138PB-TLR2 complex was performed using Gromacs v5.1.515. In the MD simulation protocol, energy minimization is carried out in stages. At 300 degrees Kelvin (constant particle number, volume, and temperature equilibrium (NVT)) and 1 bar (constant particle number, volume, and temperature (NPT) equilibrium), protein atoms and solvent molecules are equilibrated around the protein molecules by 1 ns. The forecast results showed that the HP13138PB-TLR2 temperature graph fluctuated between 299- and 301-degrees Kelvin, with a slight fluctuation range (**Figure 5C**). The results of the pressure graph showed that the fluctuation value of the HP13138PB-TLR2 was 0.68 bar (**Figure 5D**).

### 3.8 Immune simulation by the HP13138PB vaccine

**Figure 6:**
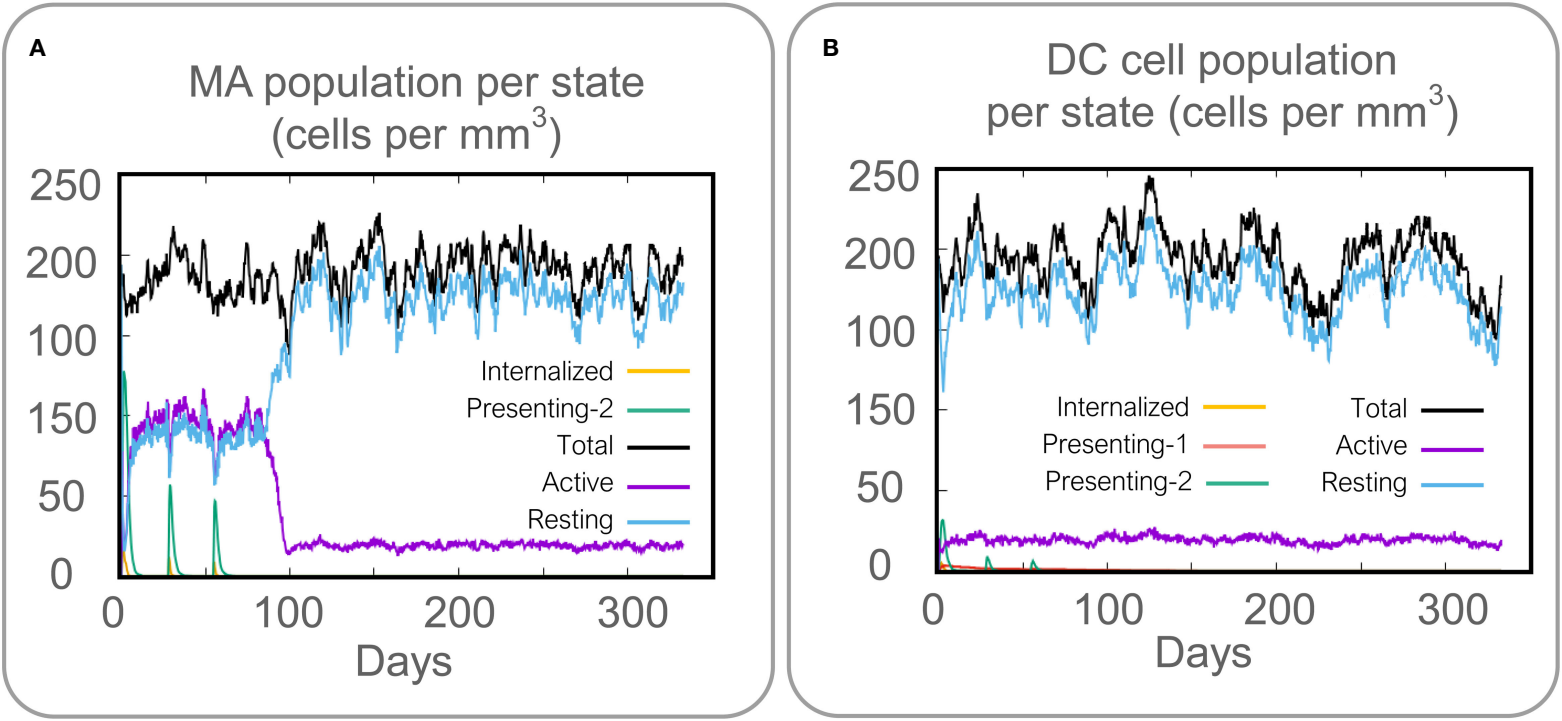
Prediction results of the C-ImmSim Server on macrophages (MA) and dendritic cells (DCs). **(A)** MA population per state (cells per mm^3^), **(B)** DC population per state (cells per mm^3^).

**Figure 7:**
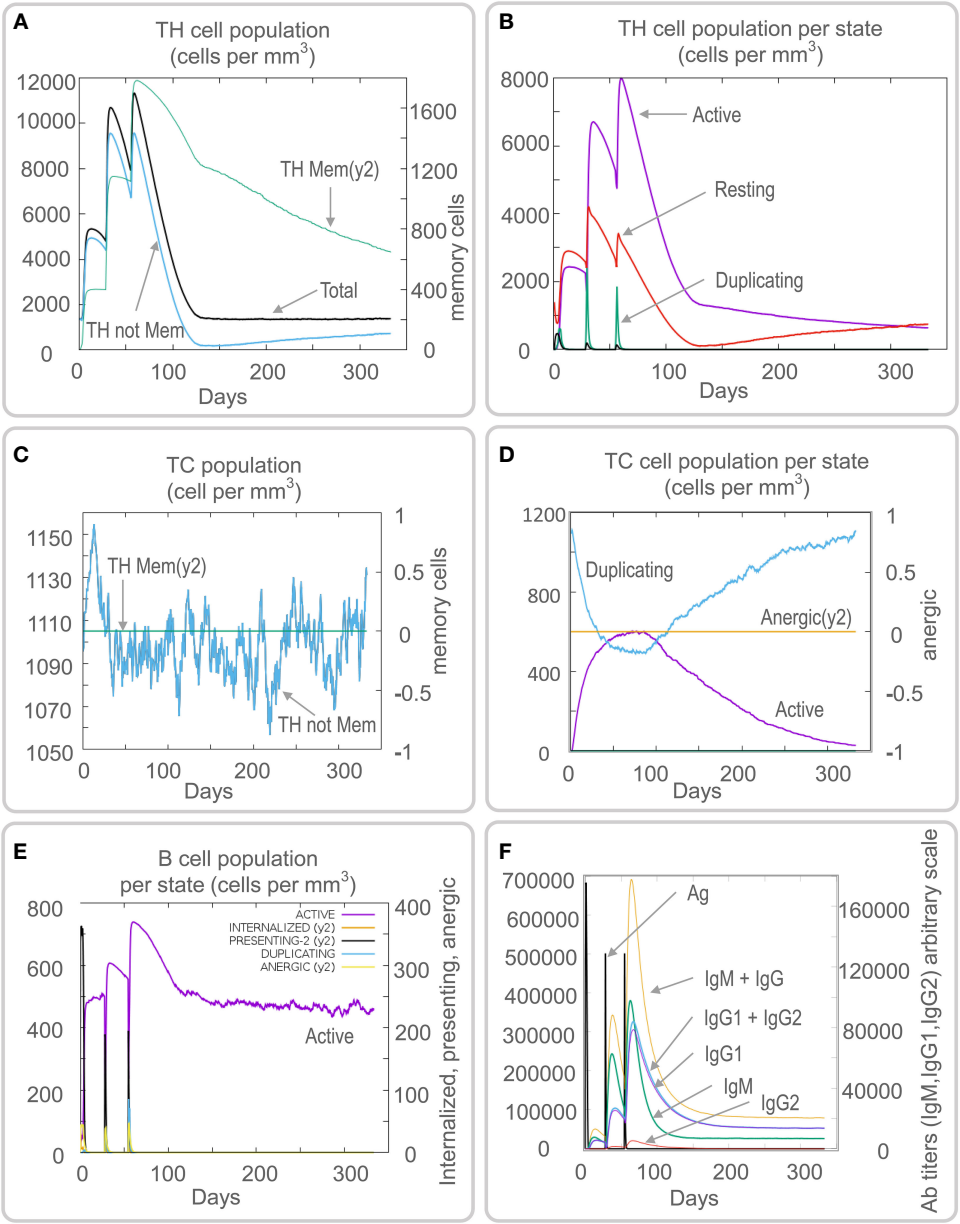
Prediction results of the C-ImmSim Server on helper T (TH) cell, cytotoxic T (TC) cell, B cell, and antibody. **(A)** TH cell population (cells per mm^3^), **(B)** TH cell population per state (cells per mm^3^), **(C)** TC cell population (cells per mm^3^), **(D)** TC cell population per state (cells per mm^3^), **(E)** B cell population per state (cells per mm^3^), **(F)** antibody titers of IgM, IgG1, and IgG2.

Vaccines can stimulate the immune cells to produce specific and non-specific immune responses that are an important way to eliminate MTB *in vivo*. In this study, we predicted the potential of the HP13138PB vaccine to stimulate an immune response in B cells, NK cells, Macrophages, CD4^+^ T cells (Th1 and Th2), and CD8^+^ T cells. It was found that the HP13138PB vaccine activated macrophages to produce phagocytosis, and the population of total Macrophages was maintained at about 200 cells/mm^3^ (**Figure 6A**). Besides, the active macrophages were held at 150 cells/mm^3^ during three immuno-simulated injections (**Figure 6A**). Similarly, DC has the strongest antigen commission capacity, and its total population was maintained at 200 cells/mm^3^ (**Figure 6B**). T cell immune response significantly eliminates MTB, of which CD4^+^ T cells are vital (52). The results showed that the population of total TH cells increased significantly in immunization simulations and peaked in the third injection, and the memory and non-memory TH cell peaks were 12,000 cells/mm^3^ and 95,000 cells/mm^3^, respectively (**Figure 7A**). In addition, the population per state of active TH cells reached 8000 cells/mm^3^ on the third injection (**Figure 7B**). The CD8^+^ T cells can kill MTB by secreting cytotoxic substances. The C-ImmSim server analysis showed that the population of non-memory TC cells peaked at 1150 cells/mm^3^ after immune injection (**Figure 7C**). Interestingly, the population per state of resting TC cells was lowest when the number of active TC cells reached a peak of 600 cells/mm^3^(**Figure 7D**). The B cells mainly produce humoral immunity *in vivo*, and the results showed that the HP13138PB vaccine could activate B cells. The population per state reached > 720 cells/mm^3^ after the third immuno-simulated injection (**Figure 7E**). Furthermore, the H113132 vaccine induced high levels of IgM and IgG antibodies, and the titers of IgM + IgG after the third injection reached 700000. Among them, the titer of IgM was >350000, accounting for a half (**Figure 7F**).

### 3.9 Plasmid construction and expression of vaccine

**Figure 8:**
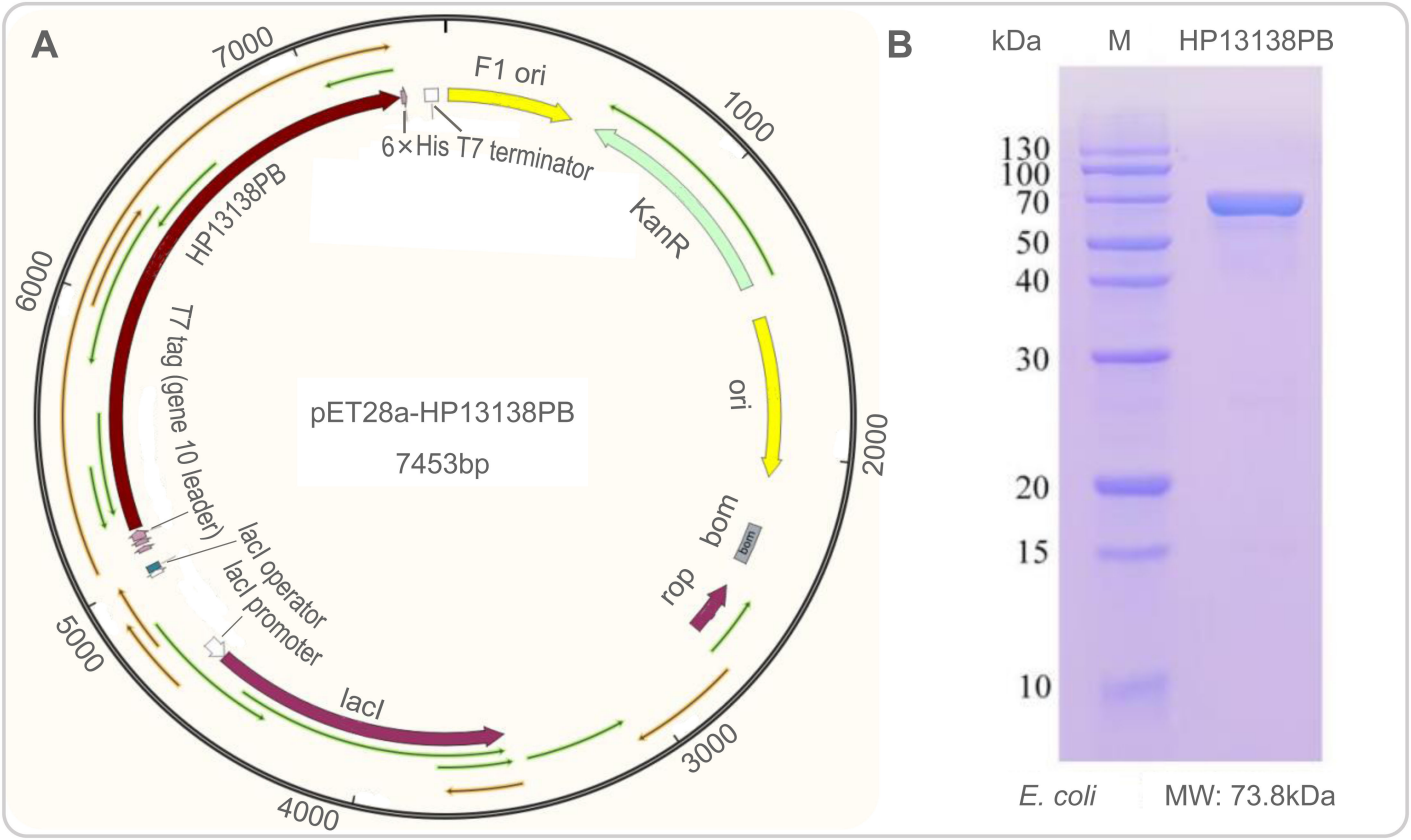
Construction of pET28a-HP13138PB recombinant plasmid *in silico* and its expression *in vitro*. **(A)** The nucleotide sequence of the HP13138PB vaccine was inserted into the arrangement of the pET28a plasmid through the restriction sites *BamHI* and *XhoI*, and the recombinant plasmid pET28a-HP13138PB had a gene length of 7453 base pairs. **(B)** The expression result of the HP13138PB vaccine in *E coli.*.

The plasmid constructed of the HP13138PB vaccine is shown in **Figure 8A**. The HP13138PB vaccine was successfully expressed in *E. coli* with a molecular weight of 73.8 kDa (**Figure 8B**).

### 3.10 Verification of the levels of cytokines induced by the HP13138PB vaccine *in silico* and *in vitro*

**Figure 9:**
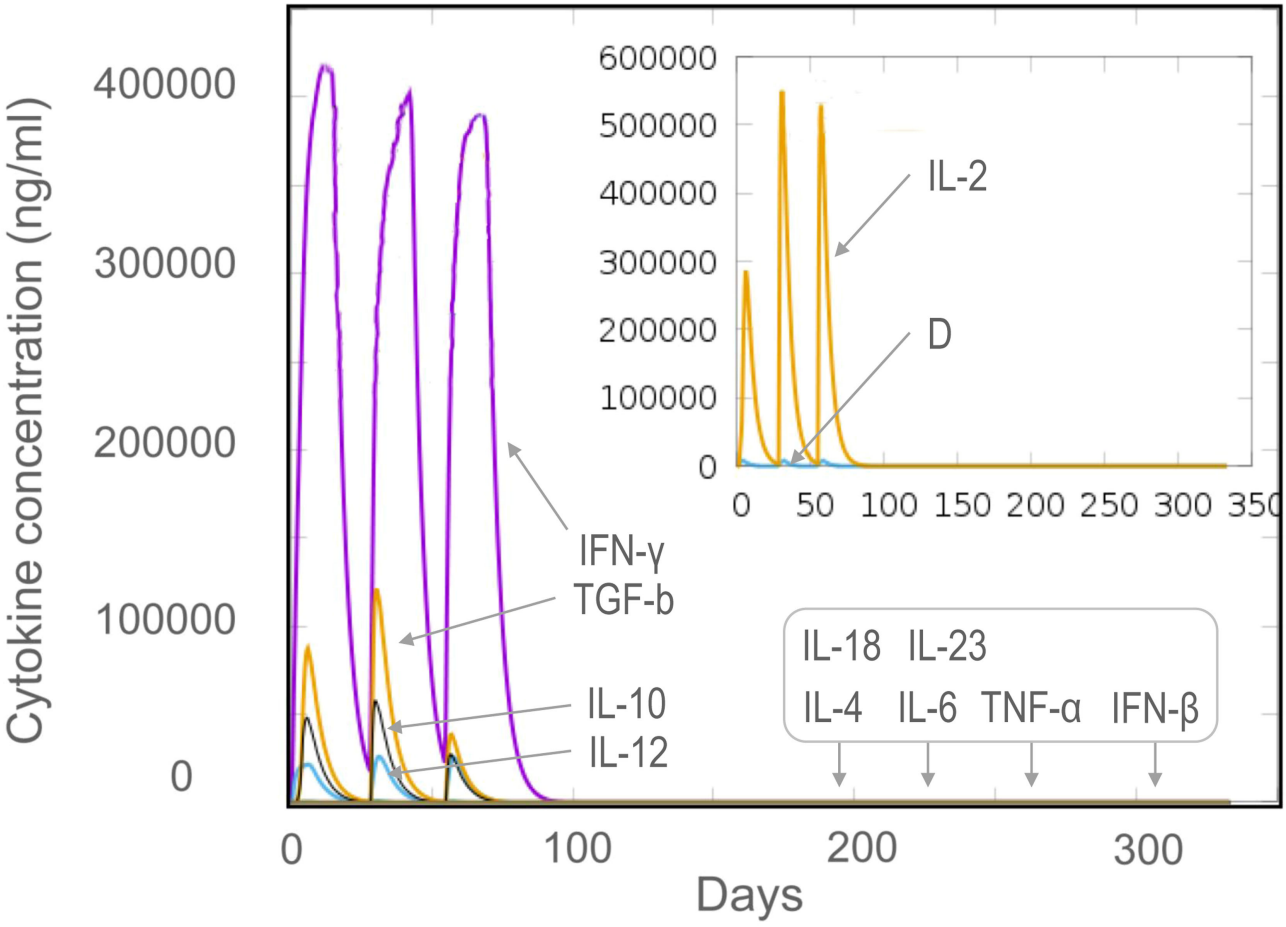
The levels of cytokines induced by the HP13138PB vaccine in the C-ImmSim Server. Three times injection of the HP13138PB vaccine was simulated in the C-ImmSim Server, and the levels of IFN-γ, IL-4, IL-12, TGF-β, TNF-α, IL-10, IL-6, IFN-β, IL-18, IL-23, and IL-2 cytokines induced by the HP13138PB vaccine were analyzed. Different cytokines were distinguished by different colors. Cytokine concentrations were expressed as ng/ml.

**Figure 10:**
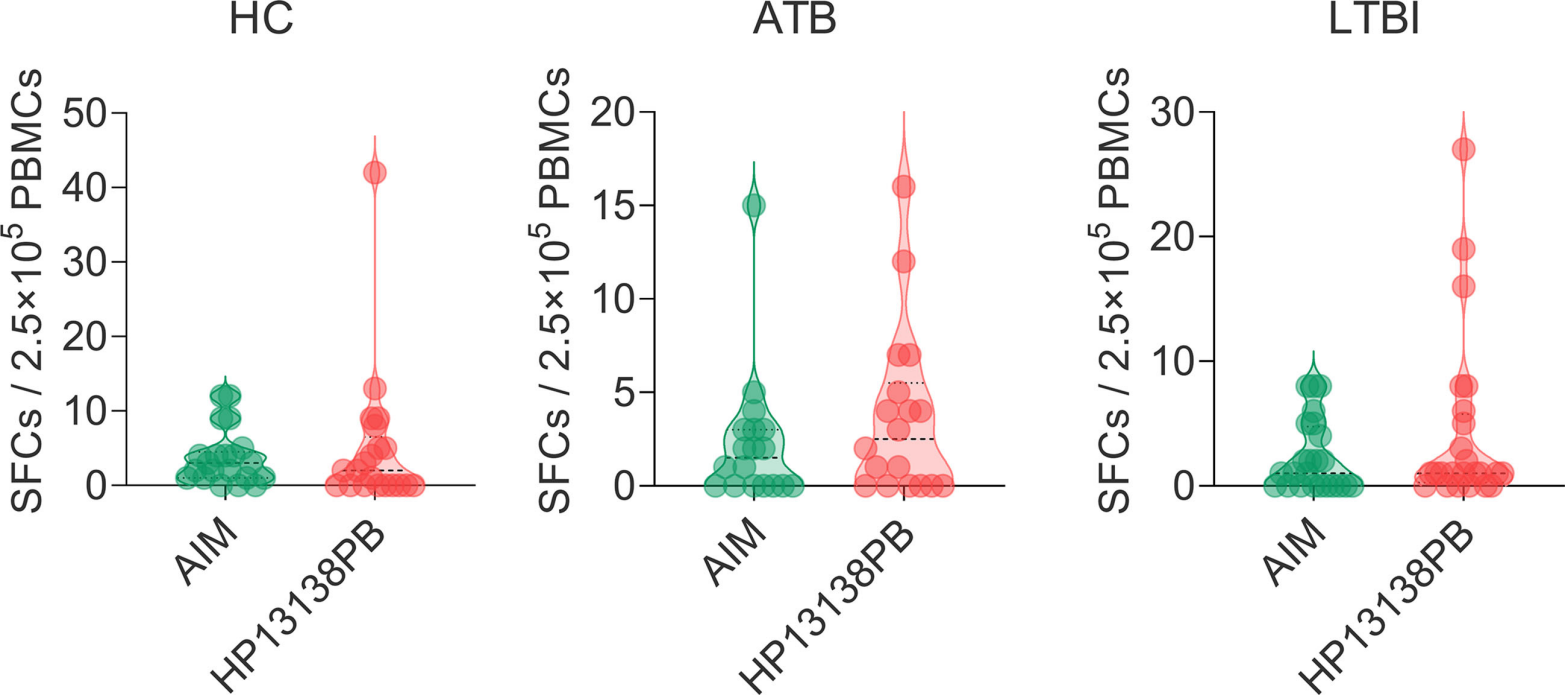
IFN-γ^+^ T lymphocytes detection with enzyme-linked immunospot assay (ELISPOT). The HP13138PB vaccine was used to stimulate the peripheral blood mononuclear cells (PBMCs) collected from health control (HC), individuals with latent tuberculosis infection (LTBI), and active tuberculosis (ATB) patients *in vitro*. The spot-forming cells (SFCs) of IFN-γ^+^ T lymphocytes were determined with a human ELISPOT kit. The data were analyzed with the Unpaired *t*-test or Mann Whitney test according to the normality. Data were shown as mean + SEM (*n* = 21, 18, and 24 in HCs, ATB patients, and LTBI volunteers, respectively). *P*<0.05 was considered significantly different. SEM, standard error of the mean.

**Figure 11:**
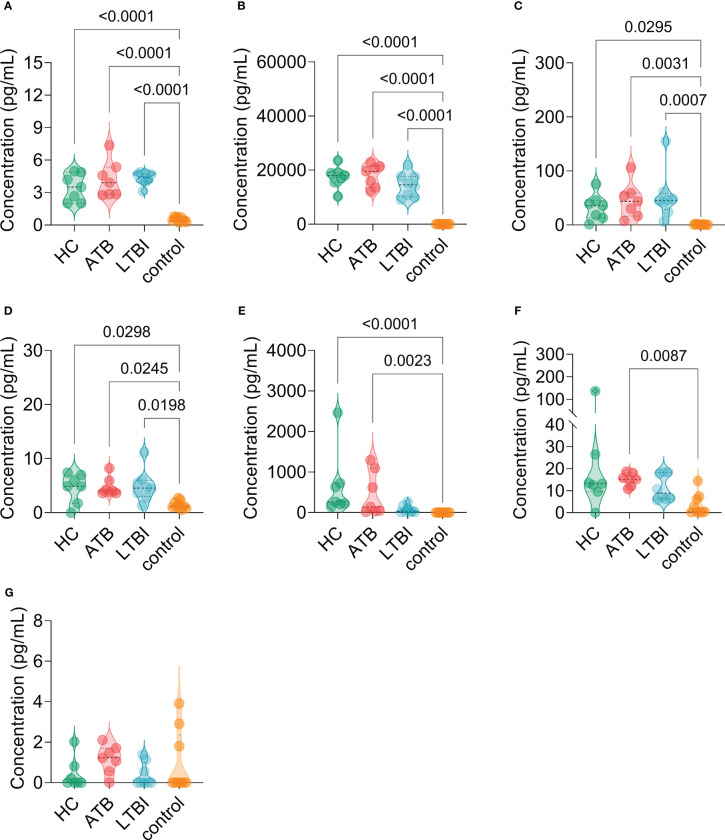
The levels of cytokines induced by the HP13138PB vaccines in peripheral blood mononuclear cells (PBMCs) from humans. The levels of interleukin-4 (IL-4, **(A)**, IL-6 **(B)**, IL-10 **(C)**, IL-17A **(D)**, tumor necrosis factor-α (TNF-α, **E**), interferon-γ (IFN-γ, **F**), and IL-2 **(G)** cytokines were detected by a human Th1/Th2/Th17 cytokine detection kit. The PBMCs collected from health control (HC, *n*=21), individuals with latent tuberculosis infection (LTBI, *n*=24), and patients with active tuberculosis (ATB, *n*=18) were stimulated with the HP13138PB vaccine *in vitro*. Furthermore, the PBMCs collected from HCs were stimulated with an AIM medium as a negative control. The differences were compared with the one-way analysis of variance (ANOVA) or Kruskal-Wallis test according to the data normality and homogeneity of variances. All data were shown as mean + SEM. *P*<0.05 was considered significantly different. SEM, standard error of the mean.

The results of the C-ImmSim server showed that the HP13138PB vaccine could significantly stimulate higher levels of IFN-γ, TGF-β, IL-2, IL-10, and IL-12 to form three peaks. The highest peaks of the IFN-γ and IL-2 were 410,000 ng/ml and 550,000 ng/ml, respectively (**Figure 9**). To assess the consistency of immune responses induced by the HP13138PB vaccine *in silico* and *in vitro*, ELISPOT and cytokines detection were performed in PBMCs collected from HCs, individuals with LTBI, and ATB patients. We found that the number of IFN^+^ T cells induced by the HP13138PB vaccine was significantly higher than that induced by the auto induction medium (AIM) medium in PBMCs obtained from HC, LTBI, and ATB patients (**Figure 10**). Furthermore, we also detected the levels of IFN-γ, IL-2, IL-4, IL-6, IL-10, TNF-α, and IL-17A cytokines in HCs, LTBI individuals, and ATB patients. The results indicated that the levels of IL-4 (**Figure 11A**), IL-6 (**Figure 11B**), IL-10 (**Figure 11C**), and IL17A (**Figure 11D**) cytokines induced by the HP13138PB vaccine in ATB, HCs, and LTBI were significantly higher than that of these cytokines induced by AIM medium in HCs. The level of TNF-α induced by the HP13138PB vaccine in ATB patients and HCs was substantially higher than that induced by the AIM medium in HCs (**Figure 11E**). Moreover, the level of IFN-γ induced by the HP13138PB vaccine in ATB patients was significantly higher than that induced by AIM medium in HCs (**Figure 11F**). There was no significant difference in the level of IL-2 between HC, ATB, LTBI, and negative controls, either HP13138PB stimulated or AIM stimulated (**Figure 11G**).

## 4 Discussion

In our previous study, we designed a Th1 peptide-based TB vaccine MP3RT, which could induce high levels of IFN-γ cytokine, CD3^+^ IFN-γ^+^ T lymphocytes, and the MP3RT-specific IgG antibody in humanized mice (49). However, the protective efficacy induced by the MP3RT vaccine was not better than that induced by the BCG vaccine in the humanized mouse model due to absence of CTL and B-cell epitopes in the MP3RT vaccine design. It has been reported that CTL peptides can be recognized by CD8^+^ T cells and produce cytotoxic factors such as granzyme B and perforin, which are essential for eliminating or killing MTB (53). Furthermore, although it is traditionally believed that B cells are not as important as T cells in the prevention of MTB infection and TB disease, increasing evidence suggests that humoral immunity plays a vital role in the fight against MTB infection (54–56).

This study selected 13 HTL epitopes, 13 CTL epitopes, and 8 B cell epitopes with highest rank, antigenicity, immunogenicity, no allergenicity, and no toxicity to construct a muti-epitopes vaccine HP13138PB. It has been reported that peptide-based vaccines are prone to degradation *in vivo*, so their induced immunogenicity and antigenicity tend to be weak and not durable (4). Previous studies indicated that TLR2 is essential in mediating immune responses to fight against MTB infection *via* TLR2-MyD88-NK-κB/IRFs-IFN-I/γ (57–59). Furthermore, antimicrobial peptides are host innate defense mediators that widely exist in plants and animals (60). Herein, TLR2 agonist PSMα4, antimicrobial peptide HBD-3, and helper peptide PADRE were added to the amino acid sequence of the HP13138PB vaccine to improve its immunogen and antigenicity. Additionally, the corresponding linkers can improve the vaccine’s expression, correct folding, and stability (61). For example, AAY liker affects structural stability through protease cleavage sites, and KK linker can maintain the immunogenicity of epitopes (62, 63). Therefore, we added the flexible linker GPGPG, rigid linker AAY, and KK linker in the structure of the HP13138PB vaccine.

The physicochemical properties of peptide-based vaccines play an essential role in their biological and immunological functions (64). For proteins with a molecular mass of less than 100,000 Da, the instability needs an index of <40, and the higher the aliphatic index, the better the thermal stability (65). We found that the HP13138PB was a vaccine characterized by 70245.98 Da of molecular weight, 33.20 (<40) of the instability index, and 79.32 of the aliphatic index, indicating that this vaccine had strong stability and was not easy to be degraded. Protein molecules must be dissolved in aqueous protein matrices to diffuse and perform biological roles *in vivo*, especially in therapeutic protein expression and purification (66). The prediction results showed that the molecular solubility of the HP13138PB vaccine was 0.55, and the GRAVY value was 0.04, suggesting that this vaccine had good solubility and hydrophilicity.

The secondary and tertiary structures are the basis for the vaccine to perform biological functions. The prediction results showed that the HP13138PB vaccine contained 31% α-helix, and 55% of amino-acid residues were expected to be exposed, and this structure is conducive to the recognition of antibodies *in vivo*. After a series of verifications of the tertiary structure of the HP13138PB vaccine, it was found that the Z-score of the model was -4.47, and the energy map showed the energy value of most amino acids was below 0. In addition, the Ramachandran chart also showed that 88.22% of amino acid residues fall in the allowable region, indicating that the vaccine’s structure was acceptable.

For an antigen that induces an immune response, binding to the receptor that produces the immune response is essential (67). Our study found that the HP13138PB vaccine had lower binding energy with TLR2 receptor. On the other hand, through molecular dynamics analysis, HP13138PB bound stably to the TLR receptor under temperature 300k and pressure of 1bar, indicating that the vaccine had strong molecular resistance to external factors and could stably exert immune effects under some conditions. These results suggest that the HP13138PB candidate may be a promising vaccine against MTB infection.

In the second part, we observed the ability of the HP13138PB vaccine to stimulate immune cells and cytokine secretion. The C-ImmSim server results showed that the HP13138PB vaccine could stimulate higher levels of immune cells (CD4^+^ T lymphocytes, CD8^+^ T lymphocytes, and B lymphocytes) antibodies (IgG and IgM). The ELISPOT results found that the HP13138PB vaccine stimulated a higher level of the IFN^+^ T lymphocyte. IFN-γ can cause apoptosis of macrophages with high bacterial loads in Stat-1 dependent manner. Conversely, IFN-γ can also promote bacterial low-load macrophage survival by inhibiting bacterial replication (68). Our previous study indicated that IFN^+^ T lymphocyte was essential for the control of MTB infection (49). Therefore, these data suggested that the HP13138PB vaccine had a good ability to trigger immune cells, especially the IFN^+^ T lymphocytes.

Furthermore, the CD4^+^ T cells can be divided into Th1, Th2, Th17, and Treg cells. The Th1 cells mainly secrete cytokines such as IFN-γ、IL-2、IL-12, and TNF-α, the Th2 cells secrete IL-4 and IL-10 cytokines, and the Th17 cells secrete IL-17 cytokine (69). In C-ImmSim server prediction, we observed an increase in the secretion of cytokines such as IFN-γ, TGF-β, IL-2, IL-10, and IL-12. Furthermore, the cytokines detection experiment showed that the HP13138PB vaccine induced significantly higher levels of IFN-γ, TNF-α, IL-2, IL-4, IL-6, IL-10, TNF-α, and IL-17A cytokines in PBMCs collected from HCs, LTBI individuals, and ATB patients. Interestingly, we found that the level of IFN-γ, IL-2 and IL-10 cytokines induced by the HP13138PB vaccine *in silico* and *in vitro* were consistent *in silico* and *in vitro*. These data suggested that the HP13138PB might be a promising TB vaccine that could generate both cellular and humoral immunity.

There are some limitations in our research: 1) In terms of vaccine design, the HP13138PB vaccine only contains TLR2 agonist but not TLR4 agonist; 2) All the immunoinformatics predictions were not verified by wet experiments, and only the important ones were verified by *in vitro* experiments, such as IFN^+^ T lymphocytes and Th1/Th2/Th17 cytokines; 3) The immunogenicity and protective efficacy of the HP13138PB vaccine in animal models were not evaluated.

## 5 Conclusion

In summary, our study showed that bioinformatics and immunoinformatics technologies have significant advantages in developing peptide-based TB vaccines. In this study, a novel peptide-based vaccine HP13138PB was designed based on HTL, CTL, and B cell epitopes predicted from 17 protective antigens of MTB. In general, the HP13138PB vaccine showed high antigenicity and immunogenicity and was easily soluble in water. Moreover, the HP13138PB vaccine can bind to TLR2 and generate an immunological response. The experiments *in vitro* showed that the HP13138PB vaccine could induce high levels of IL-2, IL-4, IL-10, and IL-17A cytokines in PBMCs collected from HCs, LTBI individuals, and ATB patients, which was consistent with the prediction results *in silico*. These findings provide evidence to evaluate the consistency between *in silico* and *in vitro* or *in vivo* experiments and also lay the foundation for the development of new TB vaccines.

## Data availability statement

The original contributions presented in the study are included in the article/**Supplementary Material**. Further inquiries can be directed to the corresponding authors.

## Ethics statement

The studies involving human participants were reviewed and approved by the Medical Ethics Committee of the Eighth Medical Center of the PLA General Hospital. The patients/participants provided their written informed consent to participate in this study.

## Author contributions

Conceptualization: WG and LW; Data curation: PC, FJ, and GW; Formal analysis: PC and WG; Funding acquisition: WG and LW; Methodology: PC, FJ, GW, JW, and YX; Software: PC and WG; Writing - original draft: PC, FJ, and GW; Writing - review and editing: WG and LW. All authors contributed to the article and approved the submitted version.

## Supplementary material

Supplementary Table 2 The full list of the HTL epitopes predicted in this study.

Supplementary Table 3 The full list of the CTL epitopes predicted in this study.

Supplementary Table 4 The full list of the B-cell epitopes predicted in this study.
