# Corrigendum: Bioinformatics analysis and consistency verification of a novel tuberculosis vaccine candidate HP13138PB

**DOI:** 10.3389/fimmu.2023.1154693

**Published:** 2023-02-08

**Authors:** Peng Cheng, Fan Jiang, Guiyuan Wang, Jie Wang, Yong Xue, Liang Wang, Wenping Gong

**Affiliations:** ^1^ Tuberculosis Prevention and Control Key Laboratory/Beijing Key Laboratory of New Techniques of Tuberculosis Diagnosis and Treatment, Senior Department of Tuberculosis, The Eighth Medical Center of PLA General Hospital, Beijing, China; ^2^ Department of Geriatrics, The Eighth Medical Center of PLA General Hospital, Beijing, China; ^3^ The Second Brigade of Cadet, Basic Medical School, Air Force Military Medical University, Xi’an, Shaanxi, China; ^4^ Hebei North University, Zhangjiakou, Hebei, China

**Keywords:** tuberculosis, epitope vaccines, immunoinformatics, immune responses, bioinformatics

In the published article, there was an error in **Figure 2A** as published. The “H13132” should be changed to “HP13138PB” in the **Figure 2A**. The corrected **Figure 2** and its caption “Figure 2. (A) The molecular solubility of the HP13138PB vaccine was predicted by the Protein-Sol server. (B) The secondary structure of the HP13138PB vaccine” appear below.

**Figure 2:**
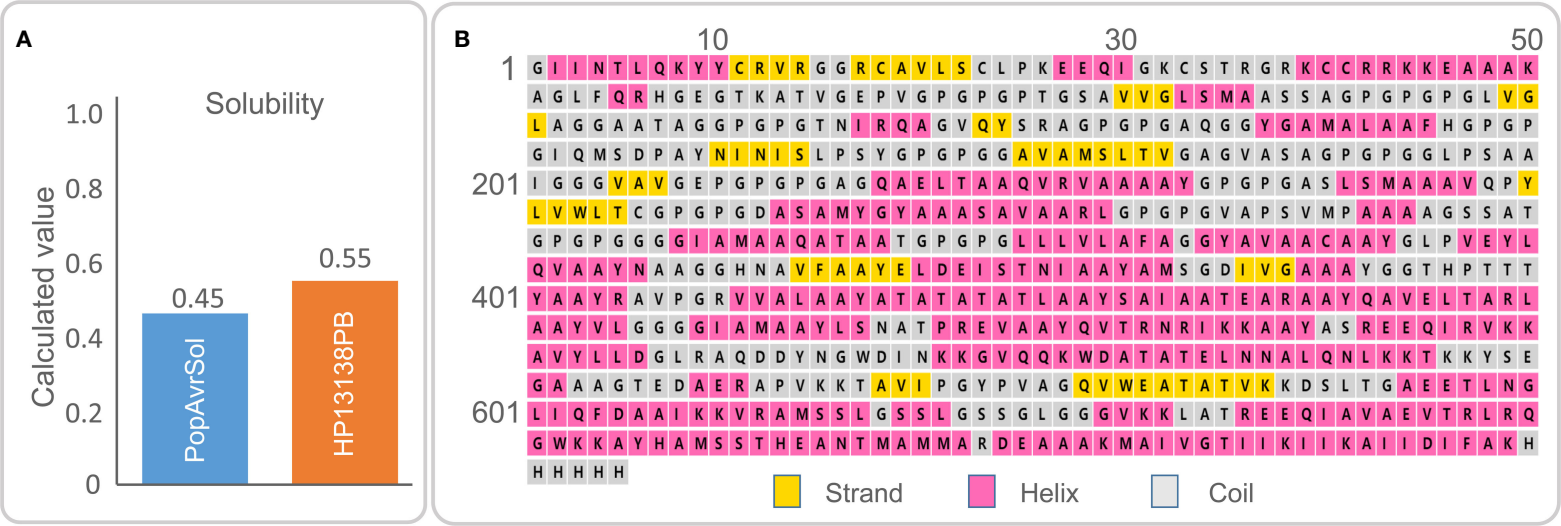
**(A)** The molecular solubility of the HP13138PB vaccine was predicted by the Protein-Sol server. **(B)** The secondary structure of the HP13138PB vaccine.

The authors apologize for this error and state that this does not change the scientific conclusions of the article in any way. The original article has been updated.

