# Supplementary material for: Bioinformatics analysis and consistency verification of a novel tuberculosis vaccine candidate HP13138PB
Source: Front Immunol. 2023 Jan 27;14:1102578. doi: 10.3389/fimmu.2023.1102578 (PMC9942524; doi:10.3389/fimmu.2023.1102578)
Supplement: Supplementary Table 2 — The full list of the HTL epitopes predicted in this study. [file Table_2.doc]

**Predicted HTL epitopes**

| **Antigen** | **Allele** | **Peptide** | **Adjusted rank** | **Antigenicity scores** | **IFN-γ scores** | **AllerTOP v.2.0*** | **Allergen FP v.1.0*** |
| --- | --- | --- | --- | --- | --- | --- | --- |
| Ag85A | HLA-DQA1*05:01/DQB1*03:01 | LVGAVGGTATAGAF | 0.24 | 0.8133 | 0.25146495 | 2 | 2 |
|  | HLA-DQA1*06:01/DQB1*03:03 | AVVGLSMAASSALTL | 0.13 | 0.7053 | 0.26519016 | 2 | 2 |
|  | HLA-DRB1*09:01 |  | 0.27 | 0.7053 | 0.26519016 | 2 | 2 |
|  | HLA-DQA1*05:01/DQB1*03:01 | GLVGAVGGTATAG | 0.37 | 1.3076 | 0.2852286 | 2 | 2 |
|  | HLA-DQA1*05:01/DQB1*03:01 | SGLVGAVGGTATAG | 0.28 | 1.2732 | 0.35392256 | 2 | 2 |
|  | HLA-DQA1*05:01/DQB1*03:01 | GLVGAVGGTATAGAF | 0.27 | 0.8397 | 0.47981667 | 2 | 2 |
|  | HLA-DQA1*05:01/DQB1*03:01 | GLVGAVGGTATAGA | 0.24 | 1.2733 | 0.52832161 | 2 | 2 |
|  | HLA-DQA1*05:01/DQB1*03:01 | LVGAVGGTATAGAFS | 0.32 | 0.8053 | 0.56918104 | 2 | 2 |
|  | HLA-DQA1*05:01/DQB1*03:01 | SGLVGAVGGTATAGAF | 0.39 | 0.8455 | 0.58228791 | 2 | 2 |
|  | HLA-DQA1*05:01/DQB1*03:01 | SGLVGAVGGTATAGA | 0.21 | 1.2452 | 0.59277922 | 2 | 2 |
|  | HLA-DQA1*06:01/DQB1*03:03 | SAVVGLSMAASSALTL | 0.13 | 0.713 | 0.67989433 | 2 | 2 |
|  | HLA-DQA1*03:01/DQB1*06:01 |  | 0.48 | 0.713 | 0.67989433 | 2 | 2 |
|  | HLA-DQA1*06:01/DQB1*03:03 | GSAVVGLSMAASSAL | 0.17 | 0.7072 | 0.73014753 | 2 | 2 |
|  | HLA-DRB1*09:01 |  | 0.34 | 0.7072 | 0.73014753 | 2 | 2 |
|  | HLA-DQA1*06:01/DQB1*03:03 | SAVVGLSMAASSA | 0.31 | 0.7507 | 0.81749766 | 2 | 2 |
|  | HLA-DQA1*06:01/DQB1*03:03 | TGSAVVGLSMAASSAL | 0.22 | 0.8133 | 0.87137816 | 2 | 2 |
|  | HLA-DQA1*06:01/DQB1*03:03 | GSAVVGLSMAASSALTL | 0.19 | 0.7896 | 0.90416742 | 2 | 2 |
|  | HLA-DQA1*06:01/DQB1*03:03 | GSAVVGLSMAASSA | 0.23 | 0.8445 | 0.91038081 | 2 | 2 |
|  | HLA-DQA1*06:01/DQB1*03:03 | PTGSAVVGLSMAASSA | 0.47 | 0.7318 | 0.91052778 | 2 | 2 |
|  | HLA-DQA1*06:01/DQB1*03:03 | TGSAVVGLSMAASSA | 0.28 | 0.9493 | 0.99130292 | 2 | 2 |
|  | HLA-DQA1*06:01/DQB1*03:03 | GSAVVGLSMAASSALT | 0.18 | 0.7454 | 1.0195956 | 2 | 2 |
|  | HLA-DQA1*06:01/DQB1*03:03 | TGSAVVGLSMAASSALTL | 0.36 | 0.8765 | 1.1333726 | 2 | 2 |
|  | HLA-DQA1*06:01/DQB1*03:03 | TGSAVVGLSMAASSALT | 0.32 | 0.8409 | 1.2023523 | 2 | 2 |
|  |  |  |  |  |  |  |  |
| Ag85B | HLA-DQA1*05:01/DQB1*03:01 | LVGLAGGAATAG | 0.33 | 1.0059 | 0.044782678 | 2 | 2 |
|  | HLA-DQA1*01:02/DQB1*06:02 | MAGSSAMILAAYHPQ | 0.23 | 0.7655 | 0.16400012 | 2 | 2 |
|  | HLA-DQA1*01:02/DQB1*06:02 | MAGSSAMILAAYHP | 0.13 | 0.7262 | 0.17760883 | 2 | 2 |
|  | HLA-DQA1*01:02/DQB1*06:02 | SMAGSSAMILAAYHP | 0.1 | 0.8015 | 0.21778925 | 2 | 2 |
|  | HLA-DQA1*01:02/DQB1*06:02 | LSMAGSSAMILAAYHPQ | 0.22 | 0.7167 | 0.361108 | 2 | 2 |
|  | HLA-DQA1*01:02/DQB1*06:02 | SMAGSSAMILAAYHPQQ | 0.39 | 0.7118 | 0.38426337 | 2 | 2 |
|  | HLA-DQA1*06:01/DQB1*03:03 | IGLSMAGSSAMILA | 0.24 | 0.7498 | 0.38426337 | 2 | 2 |
|  | HLA-DQA1*03:01/DQB1*06:01 |  | 0.44 | 0.7498 | 0.38426337 | 2 | 2 |
|  | HLA-DQA1*01:02/DQB1*06:02 | IGLSMAGSSAMILAAYH | 0.26 | 0.7095 | 0.38426337 | 2 | 2 |
|  | HLA-DQA1*06:01/DQB1*03:03 |  | 0.32 | 0.7095 | 0.38426337 | 2 | 2 |
|  | HLA-DQA1*06:01/DQB1*03:03 | AIGLSMAGSSAMIL | 0.4 | 0.8325 | 0.38599962 | 2 | 2 |
|  | HLA-DQA1*06:01/DQB1*03:03 | AAIGLSMAGSSAMIL | 0.38 | 0.7671 | 0.44011304 | 2 | 2 |
|  | HLA-DQA1*05:01/DQB1*03:01 | GLVGLAGGAATAG | 0.48 | 0.8197 | 0.52644531 | 2 | 2 |
|  | HLA-DQA1*05:01/DQB1*03:01 | PGLVGLAGGAATAG | 0.47 | 0.8884 | 0.56955554 | 2 | 2 |
|  | HLA-DQA1*06:01/DQB1*03:03 | AIGLSMAGSSAMILA | 0.28 | 0.7778 | 0.84959105 | 2 | 2 |
|  | HLA-DQA1*03:01/DQB1*06:01 |  | 0.45 | 0.7778 | 0.84959105 | 2 | 2 |
|  | HLA-DQA1*01:02/DQB1*06:02 | GLSMAGSSAMILAAY | 0.07 | 0.7257 | 0.88691652 | 2 | 2 |
|  | HLA-DQA1*03:01/DQB1*06:01 |  | 0.13 | 0.7257 | 0.88691652 | 2 | 2 |
|  | HLA-DQA1*06:01/DQB1*03:03 |  | 0.14 | 0.7257 | 0.88691652 | 2 | 2 |
|  | HLA-DQA1*03:01/DQB1*06:01 | IGLSMAGSSAMILAAYH | 0.44 | 0.7095 | 1.0068284 | 2 | 2 |
|  | HLA-DQA1*01:02/DQB1*06:02 | GLSMAGSSAMILAAYHP | 0.22 | 0.7681 | 1.0101792 | 2 | 2 |
|  | HLA-DQA1*06:01/DQB1*03:03 |  | 0.46 | 0.7681 | 1.0101792 | 2 | 2 |
|  | HLA-DQA1*03:01/DQB1*06:01 | AAIGLSMAGSSAMILAA | 0.49 | 0.7215 | 1.1091804 | 2 | 2 |
|  | HLA-DQA1*06:01/DQB1*03:03 | AIGLSMAGSSAMILAAY | 0.24 | 0.7870 | 1.1462764 | 2 | 2 |
|  | HLA-DQA1*01:02/DQB1*06:02 |  | 0.26 | 0.7870 | 1.1462764 | 2 | 2 |
|  | HLA-DRB1*09:01 |  | 0.32 | 0.7870 | 1.1462764 | 2 | 2 |
|  | HLA-DQA1*03:01/DQB1*06:01 |  | 0.36 | 0.7870 | 1.1462764 | 2 | 2 |
|  |  |  |  |  |  |  |  |
| CFP10 | HLA-DQA1*03:01/DQB1*06:01 | NIRQAGVQYSRA | 0.36 | 0.7973 | 0.71768988 | 2 | 2 |
|  | HLA-DQA1*03:01/DQB1*06:01 | TNIRQAGVQYSRA | 0.31 | 0.7348 | 0.61651125 | 2 | 2 |
|  |  |  |  |  |  |  |  |
| MPT51 | HLA-DQA1*03:01/DQB1*06:01 | AQGGYGAMALAAFH | 0.33 | 0.8205 | 0.024282193 | 2 | 2 |
|  |  |  |  |  |  |  |  |
| Mpt64 | HLA-DRB3*02:02 | DPAYNINISLPSYYP | 0.05 | 1.3183 | 0.078407167 | 2 | 2 |
|  | HLA-DRB3*02:02 | DPAYNINISLPSY | 0.12 | 1.2793 | 0.16369251 | 2 | 2 |
|  | HLA-DRB3*02:02 | PAYNINISLPSYYPD | 0.05 | 1.5020 | 0.26103359 | 2 | 2 |
|  | HLA-DRB3*02:02 | PAYNINISLPSYY | 0.03 | 1.5026 | 0.38271368 | 2 | 2 |
|  | HLA-DRB3*02:02 | AYNINISLPSYY | 0.03 | 1.4964 | 0.40067475 | 2 | 2 |
|  | HLA-DRB3*02:02 | SDPAYNINISLPSY | 0.11 | 1.1452 | 0.43666374 | 2 | 2 |
|  | HLA-DRB3*02:02 | SDPAYNINISLPSYYP | 0.06 | 1.2000 | 0.43982911 | 2 | 2 |
|  | HLA-DRB3*02:02 | DPAYNINISLPSYY | 0.03 | 1.2063 | 0.48930322 | 2 | 2 |
|  | HLA-DRB3*02:02 | DPAYNINISLPSYYPD | 0.07 | 1.2516 | 0.49281128 | 2 | 2 |
|  | HLA-DRB3*02:02 | MSDPAYNINISLPSYYP | 0.17 | 1.0854 | 0.54632885 | 2 | 2 |
|  | HLA-DRB3*02:02 | QMSDPAYNINISLPSY | 0.23 | 1.0484 | 0.70428346 | 2 | 2 |
|  | HLA-DRB3*02:02 | SDPAYNINISLPSYY | 0.05 | 1.0888 | 0.77816728 | 2 | 2 |
|  | HLA-DRB3*02:02 | IQMSDPAYNINISLPSY | 0.46 | 1.1439 | 0.7822075 | 2 | 2 |
|  | HLA-DRB3*02:02 | MSDPAYNINISLPSYY | 0.06 | 0.9757 | 0.82667295 | 2 | 2 |
|  | HLA-DRB3*02:02 | SDPAYNINISLPSYYPD | 0.17 | 1.1451 | 0.87939569 | 2 | 2 |
|  | HLA-DRB3*02:02 | QMSDPAYNINISLPSYYP | 0.38 | 1.1063 | 0.88904649 | 2 | 2 |
|  | HLA-DRB3*02:02 | MSDPAYNINISLPSYYPD | 0.38 | 1.0403 | 1.024208 | 2 | 2 |
|  | HLA-DRB3*02:02 | QMSDPAYNINISLPSYY | 0.17 | 1.0062 | 1.0888751 | 2 | 2 |
|  | HLA-DRB3*02:02 | PAYNINISLPSYYPDQ | 0.08 | 1.3614 | 2 | 2 | 2 |
|  | HLA-DRB3*02:02 | PAYNINISLPSYYPDQK | 0.2 | 1.3174 | 2 | 2 | 2 |
|  | HLA-DRB3*02:02 | DPAYNINISLPSYYPDQ | 0.2 | 1.1395 | 2 | 2 | 2 |
|  | HLA-DRB3*02:02 | SDPAYNINISLPSYYPDQ | 0.41 | 1.0470 | 2 | 2 | 2 |
|  |  |  |  |  |  |  |  |
| MTB8.4 | HLA-DQA1*06:01/DQB1*03:03 | VAMSLTVGAGVASA | 0.23 | 1.0836 | 0.076387081 | 2 | 2 |
|  | HLA-DQA1*05:01/DQB1*03:01 |  | 0.24 | 1.0836 | 0.076387081 | 2 | 2 |
|  | HLA-DQA1*03:01/DQB1*06:01 |  | 0.34 | 1.0836 | 0.076387081 | 2 | 2 |
|  | HLA-DQA1*05:01/DQB1*03:01 | AVAMSLTVGAGVASA | 0.27 | 1.0729 | 0.099654078 | 2 | 2 |
|  | HLA-DQA1*06:01/DQB1*03:03 |  | 0.28 | 1.0729 | 0.099654078 | 2 | 2 |
|  | HLA-DQA1*03:01/DQB1*06:01 |  | 0.34 | 1.0729 | 0.099654078 | 2 | 2 |
|  | HLA-DQA1*03:01/DQB1*06:01 | ALSAGVGAVAMSLTV | 0.2 | 0.9741 | 0.10142144 | 2 | 2 |
|  | HLA-DQA1*05:01/DQB1*03:01 |  | 0.27 | 0.9741 | 0.10142144 | 2 | 2 |
|  | HLA-DQA1*06:01/DQB1*03:03 |  | 0.28 | 0.9741 | 0.10142144 | 2 | 2 |
|  | HLA-DQA1*05:01/DQB1*03:01 | LSAGVGAVAMSLTVG | 0.39 | 0.9471 | 0.14452839 | 2 | 2 |
|  | HLA-DQA1*03:01/DQB1*06:01 |  | 0.46 | 0.9471 | 0.14452839 | 2 | 2 |
|  | HLA-DQA1*03:01/DQB1*06:01 | LSLTALSAGVGAVAMSL | 0.32 | 0.8207 | 0.15972067 | 2 | 2 |
|  | HLA-DQA1*05:01/DQB1*03:01 |  | 0.41 | 0.8207 | 0.15972067 | 2 | 2 |
|  | HLA-DQA1*06:01/DQB1*03:03 |  | 0.41 | 0.8207 | 0.15972067 | 2 | 2 |
|  | HLA-DQA1*05:01/DQB1*03:01 | ALSAGVGAVAMSLTVG | 0.28 | 0.9505 | 0.3275967 | 2 | 2 |
|  | HLA-DQA1*03:01/DQB1*06:01 |  | 0.35 | 0.9505 | 0.3275967 | 2 | 2 |
|  | HLA-DQA1*06:01/DQB1*03:03 | GAVAMSLTVGAGVASA | 0.45 | 0.9583 | 0.40174423 | 2 | 2 |
|  | HLA-DQA1*03:01/DQB1*06:01 | TALSAGVGAVAMSLTV | 0.18 | 0.8794 | 0.40997633 | 2 | 2 |
|  | HLA-DQA1*05:01/DQB1*03:01 |  | 0.28 | 0.8794 | 0.40997633 | 2 | 2 |
|  | HLA-DQA1*06:01/DQB1*03:03 |  | 0.28 | 0.8794 | 0.40997633 | 2 | 2 |
|  | HLA-DQA1*05:01/DQB1*03:01 | LSAGVGAVAMSLTVGA | 0.32 | 0.9498 | 0.45484551 | 2 | 2 |
|  | HLA-DQA1*05:01/DQB1*03:01 | ALSAGVGAVAMSLTVGA | 0.43 | 0.9526 | 0.59925408 | 2 | 2 |
|  | HLA-DQA1*05:01/DQB1*03:01 | TALSAGVGAVAMSLTVG | 0.43 | 0.8642 | 0.62243354 | 2 | 2 |
|  | HLA-DQA1*03:01/DQB1*06:01 |  | 0.44 | 0.8642 | 0.62243354 | 2 | 2 |
|  | HLA-DQA1*03:01/DQB1*06:01 | LTALSAGVGAVAMSLTV | 0.32 | 0.7823 | 0.77395146 | 2 | 2 |
|  | HLA-DQA1*05:01/DQB1*03:01 |  | 0.41 | 0.7823 | 0.77395146 | 2 | 2 |
|  | HLA-DQA1*06:01/DQB1*03:03 |  | 0.46 | 0.7823 | 0.77395146 | 2 | 2 |
|  |  |  |  |  |  |  |  |
| MTB32A | HLA-DQA1*05:01/DQB1*03:01 | LPSAAIGGGVAVG | 0.06 | 1.2051 | 0.034164595 | 2 | 2 |
|  | HLA-DPA1*01/DPB1*04:01 | NSRRRSLRWSWLLSVL | 0.44 | 1.4821 | 0.042552759 | 2 | 2 |
|  | HLA-DQA1*05:01/DQB1*03:01 | LPSAAIGGGVAVGE | 0.09 | 1.0985 | 0.073022294 | 2 | 2 |
|  | HLA-DQA1*05:01/DQB1*03:01 | PSAAIGGGVAVGE | 0.06 | 1.1649 | 0.13982482 | 2 | 2 |
|  | HLA-DQA1*05:01/DQB1*03:01 | GLPSAAIGGGVAVGEP | 0.18 | 0.8414 | 0.21165345 | 2 | 2 |
|  | HLA-DQA1*05:01/DQB1*03:01 | GLPSAAIGGGVAVG | 0.09 | 1.0282 | 0.27133729 | 2 | 2 |
|  | HLA-DPA1*01/DPB1*04:01 | RSLRWSWLLSVLAA | 0.24 | 1.3914 | 0.32338599 | 2 | 2 |
|  | HLA-DQA1*02:01/DQB1*05:02 |  | 0.44 | 1.3914 | 0.32338599 | 2 | 2 |
|  | HLA-DPA1*01/DPB1*04:01 | RRSLRWSWLLSVLA | 0.23 | 1.3416 | 0.35986201 | 2 | 2 |
|  | HLA-DQA1*02:01/DQB1*05:02 |  | 0.43 | 1.3416 | 0.35986201 | 2 | 2 |
|  | HLA-DQA1*05:01/DQB1*03:01 | GLPSAAIGGGVAVGE | 0.03 | 0.9452 | 0.38074847 | 2 | 2 |
|  | HLA-DPA1*01/DPB1*04:01 | RRSLRWSWLLSVLAA | 0.26 | 1.2697 | 0.38942458 | 2 | 2 |
|  | HLA-DPA1*01/DPB1*04:01 | SRRRSLRWSWLLSVLA | 0.43 | 1.3149 | 0.57734993 | 2 | 2 |
|  | HLA-DPA1*01/DPB1*04:01 | RRRSLRWSWLLSVLAA | 0.43 | 1.2614 | 0.59880927 | 2 | 2 |
|  |  |  |  |  |  |  |  |
| PPE18 | HLA-DQA1*01:02/DQB1*06:02 | GQAELTAAQVRVA | 0.3 | 1.1803 | 0.12951365 | 2 | 2 |
|  | HLA-DQA1*01:02/DQB1*06:02 | QAELTAAQVRVAA | 0.06 | 1.0634 | 0.21540075 | 2 | 2 |
|  | HLA-DQA1*03:01/DQB1*06:01 |  | 0.48 | 1.0634 | 0.21540075 | 2 | 2 |
|  | HLA-DQA1*01:02/DQB1*06:02 | GQAELTAAQVRVAA | 0.09 | 1.1007 | 0.24032333 | 2 | 2 |
|  | HLA-DQA1*01:02/DQB1*06:02 | AGQAELTAAQVRVAA | 0.19 | 1.1474 | 0.27061253 | 2 | 2 |
|  | HLA-DQA1*03:01/DQB1*06:01 | AAQVRVAAAAYETA | 0.48 | 0.7615 | 0.33511965 | 2 | 2 |
|  | HLA-DQA1*01:02/DQB1*06:02 | AELTAAQVRVAA | 0.12 | 1.0098 | 0.36090329 | 2 | 2 |
|  | HLA-DQA1*01:02/DQB1*06:02 | QAELTAAQVRVAAA | 0.01 | 1.0355 | 0.39388949 | 2 | 2 |
|  | HLA-DQA1*03:01/DQB1*06:01 |  | 0.11 | 1.0355 | 0.39388949 | 2 | 2 |
|  | HLA-DQA1*06:01/DQB1*03:03 |  | 0.48 | 1.0355 | 0.39388949 | 2 | 2 |
|  | HLA-DQA1*01:02/DQB1*06:02 | GQAELTAAQVRVAAA | 0.03 | 1.0714 | 0.42709333 | 2 | 2 |
|  | HLA-DQA1*03:01/DQB1*06:01 |  | 0.17 | 1.0714 | 0.42709333 | 2 | 2 |
|  | HLA-DQA1*01:02/DQB1*06:02 | AGQAELTAAQVRVAAA | 0.03 | 1.1165 | 0.46428381 | 2 | 2 |
|  | HLA-DQA1*03:01/DQB1*06:01 |  | 0.31 | 1.1165 | 0.46428381 | 2 | 2 |
|  | HLA-DQA1*03:01/DQB1*06:01 | MFGYAAATATATA | 0.03 | 0.7460 | 0.47074555 | 2 | 2 |
|  | HLA-DQA1*06:01/DQB1*03:03 |  | 0.11 | 0.7460 | 0.47074555 | 2 | 2 |
|  | HLA-DQA1*05:01/DQB1*03:01 |  | 0.19 | 0.7460 | 0.47074555 | 2 | 2 |
|  | HLA-DQA1*06:01/DQB1*03:03 | AATATATATLLPF | 0.17 | 0.8687 | 0.53400499 | 2 | 2 |
|  | HLA-DQA1*06:01/DQB1*03:03 | AATATATATLLPFE | 0.2 | 0.7022 | 0.5636224 | 2 | 2 |
|  | HLA-DQA1*03:01/DQB1*06:01 | AMFGYAAATATATAT | 0.02 | 0.7477 | 0.59432499 | 2 | 2 |
|  | HLA-DQA1*06:01/DQB1*03:03 |  | 0.06 | 0.7477 | 0.59432499 | 2 | 2 |
|  | HLA-DQA1*05:01/DQB1*03:01 |  | 0.15 | 0.7477 | 0.59432499 | 2 | 2 |
|  | HLA-DQA1*01:02/DQB1*06:02 | AELTAAQVRVAAA | 0.02 | 0.9841 | 0.59569189 | 2 | 2 |
|  | HLA-DQA1*03:01/DQB1*06:01 |  | 0.08 | 0.9841 | 0.59569189 | 2 | 2 |
|  | HLA-DQA1*01:02/DQB1*06:02 | QAELTAAQVRVAAAA | 0.02 | 0.9873 | 0.60719783 | 2 | 2 |
|  | HLA-DQA1*03:01/DQB1*06:01 |  | 0.1 | 0.9873 | 0.60719783 | 2 | 2 |
|  | HLA-DQA1*06:01/DQB1*03:03 |  | 0.49 | 0.9873 | 0.60719783 | 2 | 2 |
|  | HLA-DQA1*03:01/DQB1*06:01 | LTAAQVRVAAAAYETA | 0.21 | 0.7111 | 0.62600496 | 2 | 2 |
|  | HLA-DQA1*01:02/DQB1*06:02 |  | 0.33 | 0.7111 | 0.62600496 | 2 | 2 |
|  | HLA-DQA1*03:01/DQB1*06:01 | LTAAQVRVAAAAYE | 0.19 | 0.7601 | 0.63355616 | 2 | 2 |
|  | HLA-DQA1*01:02/DQB1*06:02 |  | 0.48 | 0.7601 | 0.63355616 | 2 | 2 |
|  | HLA-DQA1*01:02/DQB1*06:02 | GQAELTAAQVRVAAAA | 0.02 | 1.0242 | 0.64258282 | 2 | 2 |
|  | HLA-DQA1*03:01/DQB1*06:01 |  | 0.17 | 1.0242 | 0.64258282 | 2 | 2 |
|  | HLA-DQA1*01:02/DQB1*06:02 | ELTAAQVRVAAA | 0.03 | 1.0504 | 0.68161875 | 2 | 2 |
|  | HLA-DQA1*03:01/DQB1*06:01 |  | 0.15 | 1.0504 | 0.68161875 | 2 | 2 |
|  | HLA-DQA1*01:02/DQB1*06:02 | AGQAELTAAQVRVAAAA | 0.05 | 1.0697 | 0.68165369 | 2 | 2 |
|  | HLA-DQA1*03:01/DQB1*06:01 |  | 0.39 | 1.0697 | 0.68165369 | 2 | 2 |
|  | HLA-DQA1*03:01/DQB1*06:01 | LTAAQVRVAAAA | 0.21 | 0.8331 | 0.70791996 | 2 | 2 |
|  | HLA-DQA1*06:01/DQB1*03:03 | AATATATATLLP | 0.39 | 0.7285 | 0.71206931 | 2 | 2 |
|  | HLA-DQA1*06:01/DQB1*03:03 | GYAAATATATATLLPF | 0.05 | 0.8231 | 0.72270734 | 2 | 2 |
|  | HLA-DQA1*03:01/DQB1*06:01 |  | 0.21 | 0.8231 | 0.72270734 | 2 | 2 |
|  | HLA-DQA1*05:01/DQB1*03:01 |  | 0.35 | 0.8231 | 0.72270734 | 2 | 2 |
|  | HLA-DQA1*06:01/DQB1*03:03 | FGYAAATATATATLLPF | 0.05 | 0.7438 | 0.72901218 | 2 | 2 |
|  | HLA-DQA1*03:01/DQB1*06:01 |  | 0.19 | 0.7438 | 0.72901218 | 2 | 2 |
|  | HLA-DQA1*05:01/DQB1*03:01 |  | 0.46 | 0.7438 | 0.72901218 | 2 | 2 |
|  | HLA-DQA1*06:01/DQB1*03:03 | GYAAATATATATLLP | 0.04 | 0.7112 | 0.74458812 | 2 | 2 |
|  | HLA-DQA1*03:01/DQB1*06:01 |  | 0.17 | 0.7112 | 0.74458812 | 2 | 2 |
|  | HLA-DQA1*06:01/DQB1*03:03 | MFGYAAATATATATLLPF | 0.06 | 0.7094 | 0.80014054 | 2 | 2 |
|  | HLA-DQA1*03:01/DQB1*06:01 |  | 0.24 | 0.7094 | 0.80014054 | 2 | 2 |
|  | HLA-DQA1*03:01/DQB1*06:01 | LTAAQVRVAAAAY | 0.28 | 0.7675 | 0.81285243 | 2 | 2 |
|  | HLA-DQA1*01:02/DQB1*06:02 | QAELTAAQVRVAAAAYET | 0.06 | 0.8688 | 0.83867526 | 2 | 2 |
|  | HLA-DQA1*01:02/DQB1*06:02 | QAELTAAQVRVAAAAY | 0.02 | 0.9223 | 0.84846119 | 2 | 2 |
|  | HLA-DQA1*03:01/DQB1*06:01 |  | 0.16 | 0.9223 | 0.84846119 | 2 | 2 |
|  | HLA-DQA1*01:02/DQB1*06:02 | ELTAAQVRVAAAA | 0.02 | 0.9893 | 0.87621856 | 2 | 2 |
|  | HLA-DQA1*03:01/DQB1*06:01 |  | 0.06 | 0.9893 | 0.87621856 | 2 | 2 |
|  | HLA-DQA1*01:02/DQB1*06:02 | ELTAAQVRVAAAAYE | 0.03 | 0.8944 | 0.8889574 | 2 | 2 |
|  | HLA-DQA1*03:01/DQB1*06:01 |  | 0.12 | 0.8944 | 0.8889574 | 2 | 2 |
|  | HLA-DQA1*01:02/DQB1*06:02 | GQAELTAAQVRVAAAAY | 0.03 | 0.9605 | 0.918138 | 2 | 2 |
|  | HLA-DQA1*03:01/DQB1*06:01 |  | 0.27 | 0.9605 | 0.918138 | 2 | 2 |
|  | HLA-DQA1*01:02/DQB1*06:02 | AELTAAQVRVAAAAYE | 0.02 | 0.8552 | 0.94024149 | 2 | 2 |
|  | HLA-DQA1*03:01/DQB1*06:01 |  | 0.16 | 0.8552 | 0.94024149 | 2 | 2 |
|  | HLA-DQA1*01:02/DQB1*06:02 | GQAELTAAQVRVAAAAYE | 0.06 | 0.9422 | 0.99100041 | 2 | 2 |
|  | HLA-DQA1*01:02/DQB1*06:02 | AGQAELTAAQVRVAAAAY | 0.06 | 1.0066 | 0.99194898 | 2 | 2 |
|  | HLA-DQA1*01:02/DQB1*06:02 | AELTAAQVRVAAAAY | 0.02 | 0.8695 | 0.99768283 | 2 | 2 |
|  | HLA-DQA1*03:01/DQB1*06:01 |  | 0.1 | 0.8695 | 0.99768283 | 2 | 2 |
|  | HLA-DQA1*01:02/DQB1*06:02 | ELTAAQVRVAAAAY | 0.01 | 0.9136 | 1.0123077 | 2 | 2 |
|  | HLA-DQA1*03:01/DQB1*06:01 |  | 0.1 | 0.9136 | 1.0123077 | 2 | 2 |
|  | HLA-DQA1*06:01/DQB1*03:03 |  | 0.46 | 0.9136 | 1.0123077 | 2 | 2 |
|  | HLA-DQA1*01:02/DQB1*06:02 | AELTAAQVRVAAAAYETA | 0.18 | 0.7988 | 1.0131456 | 2 | 2 |
|  | HLA-DQA1*03:01/DQB1*06:01 |  | 0.47 | 0.7988 | 1.0131456 | 2 | 2 |
|  |  |  |  |  |  |  |  |
| PPE44 | HLA-DQA1*06:01/DQB1*03:03 | FETAFALTVPPAEVVA | 0.25 | 0.7005 | 0.030333614 | 2 | 2 |
|  | HLA-DQA1*06:01/DQB1*03:03 | FETAFALTVPPAEVV | 0.25 | 0.7060 | 0.047200693 | 2 | 2 |
|  | HLA-DQA1*03:01/DQB1*06:01 | GGAAAAVAHAVAP | 0.05 | 0.8693 | 0.10043292 | 2 | 2 |
|  | HLA-DQA1*05:01/DQB1*03:01 |  | 0.27 | 0.8693 | 0.10043292 | 2 | 2 |
|  | HLA-DQA1*06:01/DQB1*03:03 |  | 0.27 | 0.8693 | 0.10043292 | 2 | 2 |
|  | HLA-DQA1*03:01/DQB1*06:01 | GYAAASAVAARLN | 0.02 | 0.9312 | 0.12637818 | 2 | 2 |
|  | HLA-DQA1*06:01/DQB1*03:03 |  | 0.08 | 0.9312 | 0.12637818 | 2 | 2 |
|  | HLA-DQA1*05:01/DQB1*03:01 |  | 0.19 | 0.9312 | 0.12637818 | 2 | 2 |
|  | HLA-DQA1*05:01/DQB1*03:01 | APGGGAAAAVAH | 0.03 | 1.4720 | 0.15818402 | 2 | 2 |
|  | HLA-DQA1*03:01/DQB1*06:01 | AMYGYAAASAVAARL | 0.01 | 0.7444 | 0.1861288 | 2 | 2 |
|  | HLA-DQA1*06:01/DQB1*03:03 |  | 0.02 | 0.7444 | 0.1861288 | 2 | 2 |
|  | HLA-DQA1*05:01/DQB1*03:01 |  | 0.05 | 0.7444 | 0.1861288 | 2 | 2 |
|  | HLA-DRB1*09:01 |  | 0.15 | 0.7444 | 0.1861288 | 2 | 2 |
|  | HLA-DQA1*03:01/DQB1*06:01 | MYGYAAASAVAARL | 0.01 | 0.7957 | 0.19089733 | 2 | 2 |
|  | HLA-DQA1*06:01/DQB1*03:03 |  | 0.01 | 0.7957 | 0.19089733 | 2 | 2 |
|  | HLA-DQA1*05:01/DQB1*03:01 |  | 0.06 | 0.7957 | 0.19089733 | 2 | 2 |
|  | HLA-DRB1*09:01 |  | 0.39 | 0.7957 | 0.19089733 | 2 | 2 |
|  | HLA-DQA1*06:01/DQB1*03:03 | SLSMAAAVQPYLVWLTC | 0.43 | 0.7982 | 0.20170738 | 2 | 2 |
|  | HLA-DQA1*03:01/DQB1*06:01 | YGYAAASAVAARL | 0.02 | 0.8248 | 0.20183309 | 2 | 2 |
|  | HLA-DQA1*06:01/DQB1*03:03 |  | 0.02 | 0.8248 | 0.20183309 | 2 | 2 |
|  | HLA-DQA1*05:01/DQB1*03:01 |  | 0.08 | 0.8248 | 0.20183309 | 2 | 2 |
|  | HLA-DQA1*06:01/DQB1*03:03 | MGPASLSMAAAVQPYLVW | 0.15 | 0.8291 | 0.2056101 | 2 | 2 |
|  | HLA-DRB1*09:01 |  | 0.3 | 0.8291 | 0.2056101 | 2 | 2 |
|  | HLA-DQA1*06:01/DQB1*03:03 | PASLSMAAAVQPYLVWL | 0.12 | 0.9354 | 0.21786878 | 2 | 2 |
|  | HLA-DRB1*09:01 |  | 0.27 | 0.9354 | 0.21786878 | 2 | 2 |
|  | HLA-DQA1*06:01/DQB1*03:03 | ASLSMAAAVQPYLVWLT | 0.17 | 0.7222 | 0.23568725 | 2 | 2 |
|  | HLA-DQA1*03:01/DQB1*06:01 | GGAAAAVAHAVAPA | 0.01 | 0.8013 | 0.24745105 | 2 | 2 |
|  | HLA-DQA1*06:01/DQB1*03:03 |  | 0.16 | 0.8013 | 0.24745105 | 2 | 2 |
|  | HLA-DQA1*05:01/DQB1*03:01 |  | 0.18 | 0.8013 | 0.24745105 | 2 | 2 |
|  | HLA-DQA1*03:01/DQB1*06:01 | MYGYAAASAVAARLN | 0.01 | 0.8421 | 0.29374319 | 2 | 2 |
|  | HLA-DQA1*06:01/DQB1*03:03 |  | 0.02 | 0.8421 | 0.29374319 | 2 | 2 |
|  | HLA-DQA1*05:01/DQB1*03:01 |  | 0.08 | 0.8421 | 0.29374319 | 2 | 2 |
|  | HLA-DRB1*09:01 |  | 0.43 | 0.8421 | 0.29374319 | 2 | 2 |
|  | HLA-DQA1*05:01/DQB1*03:01 | AAPGGGAAAAVA | 0.03 | 1.5385 | 0.29522319 | 2 | 2 |
|  | HLA-DQA1*03:01/DQB1*06:01 | GYAAASAVAARLNP | 0.01 | 1.0049 | 0.32267528 | 2 | 2 |
|  | HLA-DQA1*06:01/DQB1*03:03 |  | 0.1 | 1.0049 | 0.32267528 | 2 | 2 |
|  | HLA-DQA1*05:01/DQB1*03:01 |  | 0.18 | 1.0049 | 0.32267528 | 2 | 2 |
|  | HLA-DQA1*05:01/DQB1*03:01 | APGGGAAAAVAHAVA | 0.02 | 1.2179 | 0.32769213 | 2 | 2 |
|  | HLA-DQA1*03:01/DQB1*06:01 |  | 0.05 | 1.2179 | 0.32769213 | 2 | 2 |
|  | HLA-DQA1*06:01/DQB1*03:03 |  | 0.35 | 1.2179 | 0.32769213 | 2 | 2 |
|  | HLA-DQA1*03:01/DQB1*06:01 | AMYGYAAASAVAARLN | 0.01 | 0.7932 | 0.35843596 | 2 | 2 |
|  | HLA-DQA1*06:01/DQB1*03:03 |  | 0.03 | 0.7932 | 0.35843596 | 2 | 2 |
|  | HLA-DQA1*05:01/DQB1*03:01 |  | 0.06 | 0.7932 | 0.35843596 | 2 | 2 |
|  | HLA-DRB1*09:01 |  | 0.3 | 0.7932 | 0.35843596 | 2 | 2 |
|  | HLA-DRB1*16:02 |  | 0.3 | 0.7932 | 0.35843596 | 2 | 2 |
|  | HLA-DQA1*03:01/DQB1*06:01 | PGGGAAAAVAHAVA | 0.05 | 1.2114 | 0.3647977 | 2 | 2 |
|  | HLA-DQA1*05:01/DQB1*03:01 |  | 0.2 | 1.2114 | 0.3647977 | 2 | 2 |
|  | HLA-DQA1*06:01/DQB1*03:03 |  | 0.37 | 1.2114 | 0.3647977 | 2 | 2 |
|  | HLA-DQA1*05:01/DQB1*03:01 | GAAPGGGAAAAVA | 0.02 | 1.6498 | 0.38485898 | 2 | 2 |
|  | HLA-DQA1*05:01/DQB1*03:01 | AAPGGGAAAAVAH | 0.02 | 1.3847 | 0.39407065 | 2 | 2 |
|  | HLA-DQA1*03:01/DQB1*06:01 | GGGAAAAVAHAVAPA | 0.04 | 1.1780 | 0.43645488 | 2 | 2 |
|  | HLA-DQA1*06:01/DQB1*03:03 |  | 0.14 | 1.1780 | 0.43645488 | 2 | 2 |
|  | HLA-DQA1*05:01/DQB1*03:01 |  | 0.27 | 1.1780 | 0.43645488 | 2 | 2 |
|  | HLA-DQA1*05:01/DQB1*03:01 | AAPGGGAAAAVAHA | 0.01 | 1.3109 | 0.45959465 | 2 | 2 |
|  | HLA-DQA1*03:01/DQB1*06:01 | GYAAASAVAARLNPL | 0.02 | 0.8418 | 0.46703886 | 2 | 2 |
|  | HLA-DQA1*06:01/DQB1*03:03 |  | 0.1 | 0.8418 | 0.46703886 | 2 | 2 |
|  | HLA-DQA1*05:01/DQB1*03:01 |  | 0.27 | 0.8418 | 0.46703886 | 2 | 2 |
|  | HLA-DQA1*03:01/DQB1*06:01 | YGYAAASAVAARLNP | 0.01 | 0.9424 | 0.47702424 | 2 | 2 |
|  | HLA-DQA1*06:01/DQB1*03:03 |  | 0.06 | 0.9424 | 0.47702424 | 2 | 2 |
|  | HLA-DQA1*05:01/DQB1*03:01 |  | 0.13 | 0.9424 | 0.47702424 | 2 | 2 |
|  | HLA-DQA1*03:01/DQB1*06:01 | MYGYAAASAVAAR | 0.02 | 0.7478 | 0.50821428 | 2 | 2 |
|  | HLA-DQA1*05:01/DQB1*03:01 |  | 0.05 | 0.7478 | 0.50821428 | 2 | 2 |
|  | HLA-DQA1*06:01/DQB1*03:03 |  | 0.05 | 0.7478 | 0.50821428 | 2 | 2 |
|  | HLA-DQA1*05:01/DQB1*03:01 | AAPGGGAAAAVAHAVA | 0.01 | 1.1678 | 0.55530471 | 2 | 2 |
|  | HLA-DQA1*03:01/DQB1*06:01 |  | 0.07 | 1.1678 | 0.55530471 | 2 | 2 |
|  | HLA-DQA1*06:01/DQB1*03:03 | ASLSMAAAVQPYLVWLTC | 0.38 | 0.7694 | 0.56423563 | 2 | 2 |
|  | HLA-DQA1*05:01/DQB1*03:01 | MGTVGGAAPGGGAAAA | 0.03 | 1.5616 | 0.57955631 | 2 | 2 |
|  | HLA-DQA1*03:01/DQB1*06:01 | SAMYGYAAASAVAARLN | 0.02 | 0.7280 | 0.59893263 | 2 | 2 |
|  | HLA-DQA1*06:01/DQB1*03:03 |  | 0.05 | 0.7280 | 0.59893263 | 2 | 2 |
|  | HLA-DQA1*05:01/DQB1*03:01 |  | 0.1 | 0.7280 | 0.59893263 | 2 | 2 |
|  | HLA-DRB1*16:02 |  | 0.29 | 0.7280 | 0.59893263 | 2 | 2 |
|  | HLA-DRB1*09:01 |  | 0.49 | 0.7280 | 0.59893263 | 2 | 2 |
|  | HLA-DQA1*03:01/DQB1*06:01 | YGYAAASAVAAR | 0.03 | 0.7757 | 0.60035564 | 2 | 2 |
|  | HLA-DQA1*05:01/DQB1*03:01 |  | 0.06 | 0.7757 | 0.60035564 | 2 | 2 |
|  | HLA-DQA1*06:01/DQB1*03:03 |  | 0.12 | 0.7757 | 0.60035564 | 2 | 2 |
|  | HLA-DQA1*05:01/DQB1*03:01 | AAPGGGAAAAVAHAV | 0.01 | 1.1958 | 0.6127018 | 2 | 2 |
|  | HLA-DQA1*03:01/DQB1*06:01 |  | 0.11 | 1.1958 | 0.6127018 | 2 | 2 |
|  | HLA-DQA1*05:01/DQB1*03:01 | GGAAPGGGAAAAVA | 0.01 | 1.7752 | 0.63741963 | 2 | 2 |
|  | HLA-DQA1*03:01/DQB1*06:01 | DASAMYGYAAASAVAARL | 0.03 | 0.7116 | 0.68087264 | 2 | 2 |
|  | HLA-DQA1*06:01/DQB1*03:03 |  | 0.06 | 0.7116 | 0.68087264 | 2 | 2 |
|  | HLA-DQA1*05:01/DQB1*03:01 |  | 0.12 | 0.7116 | 0.68087264 | 2 | 2 |
|  | HLA-DRB1*16:02 |  | 0.38 | 0.7116 | 0.68087264 | 2 | 2 |
|  | HLA-DQA1*03:01/DQB1*06:01 | YGYAAASAVAARLNPL | 0.02 | 0.7955 | 0.69654642 | 2 | 2 |
|  | HLA-DQA1*06:01/DQB1*03:03 |  | 0.05 | 0.7955 | 0.69654642 | 2 | 2 |
|  | HLA-DQA1*05:01/DQB1*03:01 |  | 0.16 | 0.7955 | 0.69654642 | 2 | 2 |
|  | HLA-DQA1*05:01/DQB1*03:01 | MGTVGGAAPGGGAAAAV | 0.02 | 1.5502 | 0.72935097 | 2 | 2 |
|  | HLA-DQA1*05:01/DQB1*03:01 | VGGAAPGGGAAAAVA | 0.01 | 1.6906 | 0.75731695 | 2 | 2 |
|  | HLA-DQA1*05:01/DQB1*03:01 | GAAPGGGAAAAVAHAVA | 0.02 | 1.2704 | 0.76517336 | 2 | 2 |
|  | HLA-DQA1*03:01/DQB1*06:01 |  | 0.1 | 1.2704 | 0.76517336 | 2 | 2 |
|  | HLA-DQA1*05:01/DQB1*03:01 | TVGGAAPGGGAAAA | 0.01 | 1.8469 | 0.7732679 | 2 | 2 |
|  | HLA-DQA1*05:01/DQB1*03:01 | TVGGAAPGGGAAAAVA | 0.01 | 1.7148 | 0.81761945 | 2 | 2 |
|  | HLA-DQA1*03:01/DQB1*06:01 | ASAMYGYAAASAVAARLN | 0.03 | 0.7457 | 0.82524798 | 2 | 2 |
|  | HLA-DQA1*06:01/DQB1*03:03 |  | 0.06 | 0.7457 | 0.82524798 | 2 | 2 |
|  | HLA-DQA1*05:01/DQB1*03:01 |  | 0.15 | 0.7457 | 0.82524798 | 2 | 2 |
|  | HLA-DRB1*16:02 |  | 0.38 | 0.7457 | 0.82524798 | 2 | 2 |
|  | HLA-DQA1*05:01/DQB1*03:01 | VGGAAPGGGAAAAV | 0.01 | 1.7974 | 0.82563928 | 2 | 2 |
|  | HLA-DQA1*05:01/DQB1*03:01 | VGGAAPGGGAAAA | 0.02 | 1.8292 | 0.82672992 | 2 | 2 |
|  | HLA-DQA1*05:01/DQB1*03:01 | MGTVGGAAPGGGAAAAVA | 0.03 | 1.4805 | 0.82707505 | 2 | 2 |
|  | HLA-DQA1*05:01/DQB1*03:01 | AMGTVGGAAPGGGAAAAV | 0.03 | 1.5188 | 0.83711781 | 2 | 2 |
|  | HLA-DQA1*05:01/DQB1*03:01 | GTVGGAAPGGGAAAAVA | 0.02 | 1.7134 | 0.90310155 | 2 | 2 |
|  | HLA-DQA1*05:01/DQB1*03:01 | VGGAAPGGGAAAAVAH | 0.01 | 1.5611 | 0.9364039 | 2 | 2 |
|  | HLA-DQA1*05:01/DQB1*03:01 | GGAAPGGGAAAAVAHAVA | 0.03 | 1.3905 | 1.0229377 | 2 | 2 |
|  | HLA-DQA1*03:01/DQB1*06:01 |  | 0.24 | 1.3905 | 1.0229377 | 2 | 2 |
|  | HLA-DQA1*05:01/DQB1*03:01 | TVGGAAPGGGAAAAVAH | 0.02 | 1.5935 | 1.0292408 | 2 | 2 |
|  | HLA-DQA1*05:01/DQB1*03:01 | GTVGGAAPGGGAAAAVAH | 0.03 | 1.5999 | 1.148739 | 2 | 2 |
|  | HLA-DQA1*05:01/DQB1*03:01 | TVGGAAPGGGAAAAVAHA | 0.03 | 1.5259 | 1.2628911 | 2 | 2 |
|  | HLA-DQA1*05:01/DQB1*03:01 | VGGAAPGGGAAAAVAHAV | 0.03 | 1.3845 | 1.2968741 | 2 | 2 |
|  |  |  |  |  |  |  |  |
| PPE68 | HLA-DQA1*03:01/DQB1*06:01 | VAPSVMPAAAAGSSAT | 0.31 | 1.0173 | 0.23735279 | 2 | 2 |
|  |  |  |  |  |  |  |  |
| RpfA | HLA-DQA1*05:01/DQB1*03:01 | AVLGGGGIAMAAQATAAT | 0.18 | 0.7919 | 1.0650068 | 2 | 2 |
|  | HLA-DQA1*03:01/DQB1*06:01 |  | 0.41 | 0.7919 | 1.0650068 | 2 | 2 |
|  | HLA-DQA1*05:01/DQB1*03:01 | TGAVLGGGGIAMAAQATA | 0.18 | 0.7212 | 1.0213486 | 2 | 2 |
|  | HLA-DQA1*05:01/DQB1*03:01 | TGAVLGGGGIAMAAQAT | 0.1 | 0.7183 | 1.0158295 | 2 | 2 |
|  | HLA-DQA1*05:01/DQB1*03:01 | GAVLGGGGIAMAAQATAA | 0.18 | 0.7836 | 0.92851612 | 2 | 2 |
|  | HLA-DQA1*03:01/DQB1*06:01 |  | 0.44 | 0.7836 | 0.92851612 | 2 | 2 |
|  | HLA-DQA1*05:01/DQB1*03:01 | GAVLGGGGIAMAAQAT | 0.07 | 0.7495 | 0.86984557 | 2 | 2 |
|  | HLA-DQA1*03:01/DQB1*06:01 |  | 0.27 | 0.7495 | 0.86984557 | 2 | 2 |
|  | HLA-DQA1*05:01/DQB1*03:01 | VLGGGGIAMAAQATAAT | 0.15 | 0.8299 | 0.82179873 | 2 | 2 |
|  | HLA-DQA1*03:01/DQB1*06:01 |  | 0.19 | 0.8299 | 0.82179873 | 2 | 2 |
|  | HLA-DQA1*06:01/DQB1*03:03 |  | 0.37 | 0.8299 | 0.82179873 | 2 | 2 |
|  | HLA-DQA1*05:01/DQB1*03:01 | GAVLGGGGIAMAAQATA | 0.1 | 0.7505 | 0.77700569 | 2 | 2 |
|  | HLA-DQA1*03:01/DQB1*06:01 |  | 0.32 | 0.7505 | 0.77700569 | 2 | 2 |
|  | HLA-DQA1*05:01/DQB1*03:01 | TGAVLGGGGIAMAAQA | 0.09 | 0.7222 | 0.66550931 | 2 | 2 |
|  | HLA-DQA1*03:01/DQB1*06:01 |  | 0.37 | 0.7222 | 0.66550931 | 2 | 2 |
|  | HLA-DQA1*05:01/DQB1*03:01 | GAVLGGGGIAMAAQ | 0.03 | 0.7514 | 0.6430047 | 2 | 2 |
|  | HLA-DQA1*05:01/DQB1*03:01 | AVLGGGGIAMAAQATAA | 0.1 | 0.7776 | 0.59581063 | 2 | 2 |
|  | HLA-DQA1*03:01/DQB1*06:01 |  | 0.19 | 0.7776 | 0.59581063 | 2 | 2 |
|  | HLA-DQA1*06:01/DQB1*03:03 |  | 0.44 | 0.7776 | 0.59581063 | 2 | 2 |
|  | HLA-DQA1*05:01/DQB1*03:01 | TGAVLGGGGIAMA | 0.11 | 0.7727 | 0.57848656 | 2 | 2 |
|  | HLA-DQA1*05:01/DQB1*03:01 | AVLGGGGIAMAAQAT | 0.03 | 0.7364 | 0.57255915 | 2 | 2 |
|  | HLA-DQA1*03:01/DQB1*06:01 |  | 0.2 | 0.7364 | 0.57255915 | 2 | 2 |
|  | HLA-DQA1*05:01/DQB1*03:01 | GAVLGGGGIAMAAQA | 0.03 | 0.7571 | 0.53964208 | 2 | 2 |
|  | HLA-DQA1*03:01/DQB1*06:01 |  | 0.27 | 0.7571 | 0.53964208 | 2 | 2 |
|  | HLA-DQA1*05:01/DQB1*03:01 | GAVLGGGGIAMAA | 0.02 | 0.8174 | 0.52899124 | 2 | 2 |
|  | HLA-DQA1*06:01/DQB1*03:03 | GGGGIAMAAQATAAT | 0.17 | 1.2534 | 0.51844941 | 2 | 2 |
|  | HLA-DQA1*03:01/DQB1*06:01 |  | 0.21 | 1.2534 | 0.51844941 | 2 | 2 |
|  | HLA-DQA1*05:01/DQB1*03:01 |  | 0.31 | 1.2534 | 0.51844941 | 2 | 2 |
|  | HLA-DQA1*05:01/DQB1*03:01 | TGAVLGGGGIAMAA | 0.03 | 0.7682 | 0.51780631 | 2 | 2 |
|  | HLA-DQA1*05:01/DQB1*03:01 | VLGGGGIAMAAQAT | 0.09 | 0.7754 | 0.49906393 | 2 | 2 |
|  | HLA-DQA1*03:01/DQB1*06:01 |  | 0.33 | 0.7754 | 0.49906393 | 2 | 2 |
|  | HLA-DQA1*05:01/DQB1*03:01 | AVLGGGGIAMAAQ | 0.02 | 0.7314 | 0.39846279 | 2 | 2 |
|  | HLA-DQA1*05:01/DQB1*03:01 | VLGGGGIAMAAQATAA | 0.09 | 0.8163 | 0.39496182 | 2 | 2 |
|  | HLA-DQA1*03:01/DQB1*06:01 |  | 0.13 | 0.8163 | 0.39496182 | 2 | 2 |
|  | HLA-DQA1*06:01/DQB1*03:03 |  | 0.22 | 0.8163 | 0.39496182 | 2 | 2 |
|  | HLA-DQA1*05:01/DQB1*03:01 | VLGGGGIAMAAQATA | 0.09 | 0.7777 | 0.31447835 | 2 | 2 |
|  | HLA-DQA1*03:01/DQB1*06:01 |  | 0.2 | 0.7777 | 0.31447835 | 2 | 2 |
|  | HLA-DQA1*05:01/DQB1*03:01 | AVLGGGGIAMAA | 0.03 | 0.8019 | 0.28755858 | 2 | 2 |
|  | HLA-DQA1*05:01/DQB1*03:01 | AVLGGGGIAMAAQA | 0.03 | 0.7421 | 0.23547819 | 2 | 2 |
|  | HLA-DQA1*03:01/DQB1*06:01 |  | 0.25 | 0.7421 | 0.23547819 | 2 | 2 |
|  | HLA-DQA1*06:01/DQB1*03:03 | GGGGIAMAAQATAA | 0.17 | 1.2718 | 0.2349098 | 2 | 2 |
|  | HLA-DQA1*03:01/DQB1*06:01 |  | 0.33 | 1.2718 | 0.2349098 | 2 | 2 |
|  | HLA-DQA1*05:01/DQB1*03:01 |  | 0.33 | 1.2718 | 0.2349098 | 2 | 2 |
|  | HLA-DQA1*06:01/DQB1*03:03 | GGGIAMAAQATAAT | 0.16 | 0.9220 | 0.13912009 | 2 | 2 |
|  | HLA-DQA1*03:01/DQB1*06:01 |  | 0.18 | 0.9220 | 0.13912009 | 2 | 2 |
| RpfB | HLA-DQA1*05:01/DQB1*03:01 | LLLVLAFAGGYAVAAC | 0.12 | 0.7437 | 0.26811457 | 2 | 2 |
|  | HLA-DQA1*03:01/DQB1*06:01 |  | 0.25 | 0.7437 | 0.26811457 | 2 | 2 |

*, AllerTOP v.2.0 and Allergen FP v.1.0 were used to predict allergenicity. 1 stands for allergenicity and 2 stands for non- allergenicity.
