# Supplementary material for: Bioinformatics analysis and consistency verification of a novel tuberculosis vaccine candidate HP13138PB
Source: Front Immunol. 2023 Jan 27;14:1102578. doi: 10.3389/fimmu.2023.1102578 (PMC9942524; doi:10.3389/fimmu.2023.1102578)
Supplement: Supplementary Table 3 — The full list of the CTL epitopes predicted in this study. [file Table_3.doc]

**Predicted CTL epitopes**

|  | peptide | Length | Immuogenicity score | Abtigenicity score | AllerTOPv.2.0* | Allergen FP v.1.0* |
| --- | --- | --- | --- | --- | --- | --- |
| Ag85A | GSAVVGLSM | 9 | 0.02032 | 1.0016 | 2 | 2 |
|  | GLPVEYLQV | 9 | 0.05492 | 0.7069 | 2 | 2 |
| Ag85B | NAAGGHNAV | 9 | 0.12765 | 1.9957 | 2 | 2 |
|  | NAAGGHNAVF | 10 | 0.16235 | 1.4758 | 2 | 2 |
|  | AAGGHNAVF | 9 | 0.12765 | 1.1841 | 2 | 2 |
|  | GLPVEYLQV | 9 | 0.05492 | 0.7069 | 2 | 2 |
| CFP10 | TAAQAAVVR | 9 | 0.03003 | 0.7677 | 2 | 2 |
|  | ELDEISTNI | 9 | 0.1108 | 0.7491 | 2 | 2 |
| MPT51 | AMSGDIVGA | 9 | 0.18192 | 1.0252 | 2 | 2 |
| Mpt63 | RTADGINYR | 9 | 0.18568 | 1.8872 | 2 | 2 |
|  | ARTADGINYR | 10 | 0.22423 | 1.8030 | 2 | 2 |
| Mpt64 | GGTHPTTTY | 9 | 0.12633 | 1.7475 | 2 | 2 |
|  | VSIAPNAGL | 9 | 0.1185 | 0.7648 | 2 | 2 |
| MTB32A | AAIGGGVAV | 9 | 0.1999 | 1.3251 | 2 | 2 |
|  | RAVPGRVVAL | 10 | 0.1822 | 1.1311 | 2 | 2 |
|  | TQDVAVLQL | 9 | 0.04866 | 1.0434 | 2 | 2 |
| PPE18 | ATATATATL | 9 | 0.1821 | 1.0012 | 2 | 2 |
| PPE44 | FLNLDVPLFV | 10 | 0.09714 | 1.3663 | 2 | 2 |
|  | SAIAATEAR | 9 | 0.26457 | 0.8366 | 2 | 2 |
| PPE68 | QAETAVNTL | 9 | 0.16574 | 0.8750 | 2 | 2 |
|  | QAVELTARL | 9 | 0.20315 | 0.7764 | 2 | 2 |
| RpfA | AVNGEPAPL | 9 | 0.1456 | 1.1393 | 2 | 2 |
|  | GLSNATPREV | 10 | 0.10743 | 1.0472 | 2 | 2 |
|  | VLGGGGIAM | 9 | 0.24518 | 0.9451 | 2 | 2 |
|  | LSNATPREV | 9 | 0.16681 | 0.7995 | 2 | 2 |
|  | EAPAETPQV | 9 | 0.09277 | 0.7822 | 2 | 2 |
|  | ASREQQIAV | 9 | 0.03089 | 0.7131 | 2 | 2 |
| RpfB | QVTRNRIKK | 9 | 0.09342 | 0.7561 | 2 | 2 |

*, AllerTOP v.2.0 and Allergen FP v.1.0 were used to predict allergenicity. 1 stands for allergenicity and 2 stands for non- allergenicity.
