# Supplementary material for: Bioinformatics analysis and consistency verification of a novel tuberculosis vaccine candidate HP13138PB
Source: Front Immunol. 2023 Jan 27;14:1102578. doi: 10.3389/fimmu.2023.1102578 (PMC9942524; doi:10.3389/fimmu.2023.1102578)
Supplement: Supplementary Table 4 — The full list of the B-cell epitopes predicted in this study. [file Table_4.docx]

**Predicted B-cell epitopes**

| protein | Sequence | Start position | Score |
| --- | --- | --- | --- |
| Ag85A | AKFLEGFVRTSNIKFQDAYN | 269 | 0.91 |
|  | RNDPLLNVGKLIANNTRVWV | 233 | 0.88 |
|  | GAQLNAMKPDLQRALGATPN | 311 | 0.86 |
| Ag85B | AVYLLDGLRAQDDYNGWDIN | 75 | 0.89 |
|  | TYKWETFLTSELPQWLSANR | 134 | 0.89 |
|  | ENFVRSSNLKFQDAYNAAGG | 270 | 0.87 |
|  | QWLSANRAVKPTGSAAIGLS | 147 | 0.85 |
| CFP10 | NFERISGDLKTQIDQVESTA | 17 | 0.88 |
|  | STNIRQAGVQYSRADEEQQQ | 73 | 0.85 |
| ESAT6 | GVQQKWDATATELNNALQNL | 53 | 0.89 |
| EspA | AGLSASSGLAHADSLASSAS | 256 | 0.9 |
|  | TKKYSEGAAAGTEDAERAPV | 357 | 0.89 |
|  | AFPGDGWLGSAADKYAGKNR | 49 | 0.86 |
|  | LESFFAGVPGLTGATSGLSQ | 229 | 0.86 |
|  | VGGALAYLVVKTLINATQLL | 145 | 0.85 |
| MPT51 | IGQAAEAMGNSRMFYNQYRS | 243 | 0.93 |
|  | NWVTAGNAMNTLAGKGISVV | 76 | 0.87 |
|  | SGDNGWGSWAPQLGAMSGDI | 275 | 0.86 |
|  | IPVAFLAGGPHAVYLLDAFN | 50 | 0.85 |
|  | GNSRMFYNQYRSVGGHNGHF | 251 | 0.85 |
| Mpt63 | TAVIPGYPVAGQVWEATATV | 63 | 0.89 |
| Mpt64 | GLDPVNYQNFAVTNDGVIFF | 181 | 0.89 |
|  | NISLPSYYPDQKSLENYIAQ | 53 | 0.88 |
|  | TRDKFLSAATSSTPREAPYE | 73 | 0.86 |
|  | VQGELSKQTGQQVSIAPNAG | 162 | 0.85 |
| MTB72f | ALGQTVQASDSLTGAEETLN | 175 | 0.89 |
|  | DSLTGAEETLNGLIQFDAAI | 184 | 0.87 |
|  | SAAIGGGVAVGEPVVAMGNS | 140 | 0.85 |
| PPE18 | VRAMSSLGSSLGSSGLGGGV | 286 | 0.89 |
|  | VDFGALPPEINSARMYAGPG | 2 | 0.85 |
|  | TPAIAVNEAEYGEMWAQDAA | 131 | 0.85 |
| PPE44 | SDVADAVLSFASPVMSAADT | 205 | 0.88 |
| PPE68 | VNTLFEKLEPMASILDPGAS | 160 | 0.91 |
|  | SATGGAAPVGAGAMGQGAQS | 317 | 0.87 |
| RpfA | AVGERVLATQGRGAWPVCGR | 97 | 0.92 |
|  | NWSINTGNGYLGGLQFTQST | 55 | 0.88 |
|  | AAPADPAPPADLAPPAPADV | 177 | 0.88 |
|  | KKLWQAIRAQDVCGNDALDS | 380 | 0.86 |
|  | LAPPAPADVAPPVELAVNDL | 188 | 0.86 |
| RpfB | LATREEQIAVAEVTRLRQGW | 330 | 0.88 |
|  | VEDPGVPGTQDVTFAVAEVN | 225 | 0.88 |
|  | AVAEVTRLRQGWGAWPVCAA | 338 | 0.87 |
|  | GNWAINTGNGYYGGVQFDQG | 295 | 0.87 |
|  | CKTVTLTVDGTAMRVTTMKS | 24 | 0.87 |
|  | NGGLRYAPRADLATREEQIA | 319 | 0.86 |
| TB10.4 | AYHAMSSTHEANTMAMMARD | 68 | 0.86 |
