# Supplementary material for: Bioinformatics analysis and consistency verification of a novel tuberculosis vaccine candidate HP13138PB
Source: Front Immunol. 2023 Jan 27;14:1102578. doi: 10.3389/fimmu.2023.1102578 (PMC9942524; doi:10.3389/fimmu.2023.1102578)
Supplement: Supplementary file 4 [file Table_1.docx]

**Table S1. The basic information about selected vaccine candidates of *M. tuberculosis.***

| Protein Name ^a^ | Accession No. ^b^ | Identifier ^a^ | | | Gene Name ^a^ | Length (aa)^a^ | | Function ^a^ | Production ^a^ | Group ^c^ | References ^d^ |
| --- | --- | --- | --- | --- | --- | --- | --- | --- | --- | --- | --- |
| Ag85A | CCP46633 | | Rv3804c | *fbpA* | | 338 | Involved in cell wall mycoloylation | | Secreted antigen 85-a FbpA | II | [1, 2] |
| Ag85B | CCP44652 | | Rv1886c | *fbpB* | | 325 | Involved in cell wall mycoloylation | | Secreted antigen 85-B FbpB (85B) | I | [3, 4] |
| CFP10 | CCP46703 | | Rv3874 | *EsxB* | | 100 | Unknown | | 10 kDa culture filtrate antigen EsxB (CFP10) | NA | [2, 4, 5] |
| ESAT6 | CCP46704 | | Rv3875 | *esxA* | | 95 | Unknown | | 6 kDa early secretory antigenic target EsxA (ESAT-6) | III | [2, 4-6] |
| EspA | CCP46439 | | Rv3616c | *espA* | | 392 | Unknown | | ESX-1 secretion-associated protein A, EspA | NA | [7, 8] |
| Mpt51 | CCP46632 | | Rv3803c | *fbpD* | | 299 | Involved in cell wall mycoloylation | | Secreted MPT51/MPB51 antigen protein FbpD | NA | [9-13] |
| Mpt63 | CCP44693 | | Rv1926c | *mpt63/mpb63* | | 159 | Immunogenic protein Mpt63 | | Predicted possible vaccine candidate | III | [14-18] |
| Mpt64 | CCP44749 | | Rv1980c | *mpt64/mpb64* | | 228 | Immunogenic protein Mpt64 | | Predicted possible vaccine candidate | II | [19-23] |
| Mtb8.4 | CCP43930 | | Rv1174c | *TB8.4* | | 110 | Low molecular weight T-cell antigen TB8.4 | | Predicted to be an outer membrane protein and possible vaccine candidate | II | [22, 24-26] |
| Mtb32a | Rv0125 | | Rv0125 | *pepA* | | 355 | Unknown | | Probable serine protease PepA (serine proteinase) (MTB32A) | NA | [27-29] |
| PPE18 | CCP43952 | | Rv1196 | *PPE18* | | 391 | PPE family protein PPE18 | | Member of the Mycobacterium tuberculosis PPE family | NA | [8, 30-33] |
| PPE44 | CCP45569 | | Rv2770c | *PPE44* | | 382 | PPE family protein PPE44 | | Member of the Mycobacterium tuberculosis PPE family | NA | [34-37] |
| PPE68 | CCP46702 | | Rv3873 | *PPE68* | | 368 | PPE family protein PPE68 | | A peptide-based vaccine candidate | I | [38-43] |
| RpfA | CCP43615 | | Rv0867c | *rpfA* | | 407 | Possible resuscitation-promoting factor RpfA | | Predicted possible vaccine candidate | I | [44-46] |
| RpfB | CCP43759 | | Rv1009 | *rpfB* | | 362 | Probable resuscitation-promoting factor RpfB | | Predicted possible vaccine candidate | I | [44, 46-51] |
| RpfE | CCP45243 | | Rv2450c | *rpfE* | | 172 | Probable resuscitation-promoting factor RpfE | | Predicted possible vaccine candidate | I | [52-55] |
| TB10.4 | CCP43018 | | Rv0288 | *esxH/cfp7/TB10.4* | | 96 | Low molecular weight protein antigen 7 EsxH | | Predicted possible vaccine candidate | I | [56-58] |

a The Gene name, Identifier, Length (aa), Function, and Production information are based on the data deposited at the Mycobrowser database (https://mycobrowser.epfl.ch/genes). Data were retrieved 23 Mar, 2021.

b The Accession No was obtained from the Protein database of NCBI (<https://www.ncbi.nlm.nih.gov/protein>). Data were retrieved 23 Mar, 2021.

c The group is based on a previous study (See Zvi et al., 2008). The antigens are sorted by the qualitative score (Qual Total) and subsequently by the quantitative score (Quant Total). Group I includes all antigens with a qualitative score 8 and above, provided that the quantitative score is not lower than 12. The rest of the antigens having a qualitative score of 8 and those having a qualitative score of 7 and a quantitative score not lower than 9 were clustered into Group II. Group III included antigens with qualitative scores of 7 (and quantitative score of 8) and 6 (with a quantitative scores 9 and up).

d The references of previously selected proteins to act as TB vaccine candidates.

NA, not available.

[1] Li W, Li M, Deng G, Zhao L, Liu X, Wang Y. Prime-boost vaccination with Bacillus Calmette Guerin and a recombinant adenovirus co-expressing CFP10, ESAT6, Ag85A and Ag85B of Mycobacterium tuberculosis induces robust antigen-specific immune responses in mice. Molecular medicine reports. 2015;12:3073-80.

[2] Tkachuk AP, Bykonia EN, Popova LI, Kleymenov DA, Semashko MA, Chulanov VP, et al. Safety and Immunogenicity of the GamTBvac, the Recombinant Subunit Tuberculosis Vaccine Candidate: A Phase II, Multi-Center, Double-Blind, Randomized, Placebo-Controlled Study. Vaccines (Basel). 2020;8.

[3] Bekker LG, Dintwe O, Fiore-Gartland A, Middelkoop K, Hutter J, Williams A, et al. A phase 1b randomized study of the safety and immunological responses to vaccination with H4:IC31, H56:IC31, and BCG revaccination in Mycobacterium tuberculosis-uninfected adolescents in Cape Town, South Africa. EClinicalMedicine. 2020;21:100313.

[4] Wang C, Lu J, Du W, Wang G, Li X, Shen X, et al. Ag85b/ESAT6-CFP10 adjuvanted with aluminum/poly-IC effectively protects guinea pigs from latent mycobacterium tuberculosis infection. Vaccine. 2019;37:4477-84.

[5] Aguilo N, Gonzalo-Asensio J, Alvarez-Arguedas S, Marinova D, Gomez AB, Uranga S, et al. Reactogenicity to major tuberculosis antigens absent in BCG is linked to improved protection against Mycobacterium tuberculosis. Nat Commun. 2017;8:16085.

[6] Aagaard C, Hoang T, Dietrich J, Cardona PJ, Izzo A, Dolganov G, et al. A multistage tuberculosis vaccine that confers efficient protection before and after exposure. Nature medicine. 2011;17:189-94.

[7] Groschel MI, Sayes F, Shin SJ, Frigui W, Pawlik A, Orgeur M, et al. Recombinant BCG Expressing ESX-1 of Mycobacterium marinum Combines Low Virulence with Cytosolic Immune Signaling and Improved TB Protection. Cell reports. 2017;18:2752-65.

[8] Mustafa AS, Skeiky YA, Al-Attiyah R, Alderson MR, Hewinson RG, Vordermeier HM. Immunogenicity of Mycobacterium tuberculosis antigens in Mycobacterium bovis BCG-vaccinated and M. bovis-infected cattle. Infection and immunity. 2006;74:4566-72.

[9] Wang LX, Nagata T, Tsujimura K, Uchijima M, Seto S, Koide Y. Identification of HLA-DR4-restricted T-cell epitope on MPT51 protein, a major secreted protein derived from Mycobacterium tuberculosis using MPT51 overlapping peptides screening and DNA vaccination. Vaccine. 2010;28:2026-31.

[10] de Sousa EM, da Costa AC, Trentini MM, de Araujo Filho JA, Kipnis A, Junqueira-Kipnis AP. Immunogenicity of a fusion protein containing immunodominant epitopes of Ag85C, MPT51, and HspX from Mycobacterium tuberculosis in mice and active TB infection. PloS one. 2012;7:e47781.

[11] da Costa AC, Costa-Junior Ade O, de Oliveira FM, Nogueira SV, Rosa JD, Resende DP, et al. A new recombinant BCG vaccine induces specific Th17 and Th1 effector cells with higher protective efficacy against tuberculosis. PloS one. 2014;9:e112848.

[12] Suzuki M, Aoshi T, Nagata T, Koide Y. Identification of murine H2-Dd- and H2-Ab-restricted T-cell epitopes on a novel protective antigen, MPT51, of Mycobacterium tuberculosis. Infection and immunity. 2004;72:3829-37.

[13] Miki K, Nagata T, Tanaka T, Kim YH, Uchijima M, Ohara N, et al. Induction of protective cellular immunity against Mycobacterium tuberculosis by recombinant attenuated self-destructing Listeria monocytogenes strains harboring eukaryotic expression plasmids for antigen 85 complex and MPB/MPT51. Infection and immunity. 2004;72:2014-21.

[14] Lee BY, Horwitz MA. T-cell epitope mapping of the three most abundant extracellular proteins of Mycobacterium tuberculosis in outbred guinea pigs. Infection and immunity. 1999;67:2665-70.

[15] Caccamo N, Barera A, Di Sano C, Meraviglia S, Ivanyi J, Hudecz F, et al. Cytokine profile, HLA restriction and TCR sequence analysis of human CD4+ T clones specific for an immunodominant epitope of Mycobacterium tuberculosis 16-kDa protein. Clinical and experimental immunology. 2003;133:260-6.

[16] Manca C, Lyashchenko K, Wiker HG, Usai D, Colangeli R, Gennaro ML. Molecular cloning, purification, and serological characterization of MPT63, a novel antigen secreted by Mycobacterium tuberculosis. Infection and immunity. 1997;65:16-23.

[17] Harth G, Lee BY, Horwitz MA. High-level heterologous expression and secretion in rapidly growing nonpathogenic mycobacteria of four major Mycobacterium tuberculosis extracellular proteins considered to be leading vaccine candidates and drug targets. Infection and immunity. 1997;65:2321-8.

[18] Mustafa AS. Th1 cell reactivity and HLA-DR binding prediction for promiscuous recognition of MPT63 (Rv1926c), a major secreted protein of Mycobacterium tuberculosis. Scandinavian journal of immunology. 2009;69:213-22.

[19] Mustafa AS, Shaban F. Mapping of Th1-cell epitope regions of Mycobacterium tuberculosis protein MPT64 (Rv1980c) using synthetic peptides and T-cell lines from M. tuberculosis-infected healthy humans. Med Princ Pract. 2010;19:122-8.

[20] Oettinger T, Holm A, Haslov K. Characterization of the delayed type hypersensitivity-inducing epitope of MPT64 from Mycobacterium tuberculosis. Scandinavian journal of immunology. 1997;45:499-503.

[21] Oettinger T, Holm A, Mtoni IM, Andersen AB, Hasloov K. Mapping of the delayed-type hypersensitivity-inducing epitope of secreted protein MPT64 from Mycobacterium tuberculosis. Infection and immunity. 1995;63:4613-8.

[22] Liu X, Peng J, Hu L, Luo Y, Niu H, Bai C, et al. A multistage mycobacterium tuberculosis subunit vaccine LT70 including latency antigen Rv2626c induces long-term protection against tuberculosis. Human vaccines & immunotherapeutics. 2016;12:1670-7.

[23] Luo Y, Jiang W, Da Z, Wang B, Hu L, Zhang Y, et al. Subunit vaccine candidate AMM down-regulated the regulatory T cells and enhanced the protective immunity of BCG on a suitable schedule. Scandinavian journal of immunology. 2012;75:293-300.

[24] Evans JT, Ward JR, Kern J, Johnson ME. A single vaccination with protein-microspheres elicits a strong CD8 T-cell-mediated immune response against Mycobacterium tuberculosis antigen Mtb8.4. Vaccine. 2004;22:1964-72.

[25] Li H, Li R, Zhong S, Ren H. [Plasmid encoding human IL-12 improve protective efficacy of Mtb8.4 gene vaccine with signal sequence against infection of Mycobacterium Tuberculosis]. Xi bao yu fen zi mian yi xue za zhi = Chinese journal of cellular and molecular immunology. 2007;23:291-4.

[26] Luo Y, Wang B, Hu L, Yu H, Da Z, Jiang W, et al. Fusion protein Ag85B-MPT64(190-198)-Mtb8.4 has higher immunogenicity than Ag85B with capacity to boost BCG-primed immunity against Mycobacterium tuberculosis in mice. Vaccine. 2009;27:6179-85.

[27] Bibi S, Ullah I, Zhu B, Adnan M, Liaqat R, Kong WB, et al. In silico analysis of epitope-based vaccine candidate against tuberculosis using reverse vaccinology. Sci Rep. 2021;11:1249.

[28] Skeiky YA, Alderson MR, Ovendale PJ, Guderian JA, Brandt L, Dillon DC, et al. Differential immune responses and protective efficacy induced by components of a tuberculosis polyprotein vaccine, Mtb72F, delivered as naked DNA or recombinant protein. Journal of immunology (Baltimore, Md : 1950). 2004;172:7618-28.

[29] Skeiky YA, Lodes MJ, Guderian JA, Mohamath R, Bement T, Alderson MR, et al. Cloning, expression, and immunological evaluation of two putative secreted serine protease antigens of Mycobacterium tuberculosis. Infection and immunity. 1999;67:3998-4007.

[30] Bhat KH, Ahmed A, Kumar S, Sharma P, Mukhopadhyay S. Role of PPE18 protein in intracellular survival and pathogenicity of Mycobacterium tuberculosis in mice. PloS one. 2012;7:e52601.

[31] Hebert AM, Talarico S, Yang D, Durmaz R, Marrs CF, Zhang L, et al. DNA polymorphisms in the pepA and PPE18 genes among clinical strains of Mycobacterium tuberculosis: implications for vaccine efficacy. Infection and immunity. 2007;75:5798-805.

[32] Homolka S, Ubben T, Niemann S. High Sequence Variability of the ppE18 Gene of Clinical Mycobacterium tuberculosis Complex Strains Potentially Impacts Effectivity of Vaccine Candidate M72/AS01E. PloS one. 2016;11:e0152200.

[33] Mortier MC, Jongert E, Mettens P, Ruelle JL. Sequence conservation analysis and in silico human leukocyte antigen-peptide binding predictions for the Mtb72F and M72 tuberculosis candidate vaccine antigens. BMC immunology. 2015;16:63.

[34] Cuccu B, Freer G, Genovesi A, Garzelli C, Rindi L. Identification of a human immunodominant T-cell epitope of mycobacterium tuberculosis antigen PPE44. BMC microbiology. 2011;11:167.

[35] Rindi L, Peroni I, Lari N, Bonanni D, Tortoli E, Garzelli C. Variation of the expression of Mycobacterium tuberculosis ppe44 gene among clinical isolates. FEMS immunology and medical microbiology. 2007;51:381-7.

[36] Moradi B, Sankian M, Amini Y, Meshkat Z. Construction of a Novel DNA Vaccine Candidate Encoding an HspX-PPE44-EsxV Fusion Antigen of Mycobacterium tuberculosis. Rep Biochem Mol Biol. 2016;4:89-97.

[37] Romano M, Rindi L, Korf H, Bonanni D, Adnet PY, Jurion F, et al. Immunogenicity and protective efficacy of tuberculosis subunit vaccines expressing PPE44 (Rv2770c). Vaccine. 2008;26:6053-63.

[38] Mustafa AS. Characterization of a cross-reactive, immunodominant and HLA-promiscuous epitope of Mycobacterium tuberculosis-specific major antigenic protein PPE68. PloS one. 2014;9:e103679.

[39] Jiang Y, Wei J, Liu H, Li G, Guo Q, Qiu Y, et al. Polymorphisms in the PE35 and PPE68 antigens in Mycobacterium tuberculosis strains may affect strain virulence and reflect ongoing immune evasion. Molecular medicine reports. 2016;13:947-54.

[40] Tiwari B, Soory A, Raghunand TR. An immunomodulatory role for the Mycobacterium tuberculosis region of difference 1 locus proteins PE35 (Rv3872) and PPE68 (Rv3873). The FEBS journal. 2014;281:1556-70.

[41] Christy AJ, Dharman K, Dhandapaani G, Palaniyandi K, Gupta UD, Gupta P, et al. Epitope based recombinant BCG vaccine elicits specific Th1 polarized immune responses in BALB/c mice. Vaccine. 2012;30:1364-70.

[42] Mustafa AS, Al-Attiyah R, Hanif SN, Shaban FA. Efficient testing of large pools of Mycobacterium tuberculosis RD1 peptides and identification of major antigens and immunodominant peptides recognized by human Th1 cells. Clinical and vaccine immunology : CVI. 2008;15:916-24.

[43] Hanif SN, Al-Attiyah R, Mustafa AS. DNA vaccine constructs expressing Mycobacterium tuberculosis-specific genes induce immune responses. Scandinavian journal of immunology. 2010;72:408-15.

[44] Russell-Goldman E, Xu J, Wang X, Chan J, Tufariello JM. A Mycobacterium tuberculosis Rpf double-knockout strain exhibits profound defects in reactivation from chronic tuberculosis and innate immunity phenotypes. Infection and immunity. 2008;76:4269-81.

[45] Rickman L, Scott C, Hunt DM, Hutchinson T, Menendez MC, Whalan R, et al. A member of the cAMP receptor protein family of transcription regulators in Mycobacterium tuberculosis is required for virulence in mice and controls transcription of the rpfA gene coding for a resuscitation promoting factor. Mol Microbiol. 2005;56:1274-86.

[46] Zhu W, Plikaytis BB, Shinnick TM. Resuscitation factors from mycobacteria: homologs of Micrococcus luteus proteins. Tuberculosis (Edinburgh, Scotland). 2003;83:261-9.

[47] Romano M, Aryan E, Korf H, Bruffaerts N, Franken CL, Ottenhoff TH, et al. Potential of Mycobacterium tuberculosis resuscitation-promoting factors as antigens in novel tuberculosis sub-unit vaccines. Microbes and infection. 2012;14:86-95.

[48] Kim JS, Kim WS, Choi HG, Jang B, Lee K, Park JH, et al. Mycobacterium tuberculosis RpfB drives Th1-type T cell immunity via a TLR4-dependent activation of dendritic cells. J Leukoc Biol. 2013;94:733-49.

[49] Mattos AM, Chaves AS, Franken KL, Figueiredo BB, Ferreira AP, Ottenhoff TH, et al. Detection of IgG1 antibodies against Mycobacterium tuberculosis DosR and Rpf antigens in tuberculosis patients before and after chemotherapy. Tuberculosis (Edinburgh, Scotland). 2016;96:65-70.

[50] Ma J, Tian M, Fan X, Yu Q, Jing Y, Wang W, et al. Mycobacterium tuberculosis multistage antigens confer comprehensive protection against pre- and post-exposure infections by driving Th1-type T cell immunity. Oncotarget. 2016;7:63804-15.

[51] Fan A, Jian W, Shi C, Ma Y, Wang L, Peng D, et al. Production and characterization of monoclonal antibody against Mycobacterium tuberculosis RpfB domain. Hybridoma (Larchmt). 2010;29:327-32.

[52] Choi HG, Kim WS, Back YW, Kim H, Kwon KW, Kim JS, et al. Mycobacterium tuberculosis RpfE promotes simultaneous Th1- and Th17-type T-cell immunity via TLR4-dependent maturation of dendritic cells. Eur J Immunol. 2015;45:1957-71.

[53] Xin Q, Niu H, Li Z, Zhang G, Hu L, Wang B, et al. Subunit vaccine consisting of multi-stage antigens has high protective efficacy against Mycobacterium tuberculosis infection in mice. PloS one. 2013;8:e72745.

[54] Oksanen KE, Myllymaki H, Ahava MJ, Makinen L, Parikka M, Ramet M. DNA vaccination boosts Bacillus Calmette-Guerin protection against mycobacterial infection in zebrafish. Developmental and comparative immunology. 2016;54:89-96.

[55] Xue Y, Bai Y, Gao X, Jiang H, Wang L, Gao H, et al. Expression, purification and characterization of Mycobacterium tuberculosis RpfE protein. Journal of biomedical research. 2012;26:17-23.

[56] Billeskov R, Grandal MV, Poulsen C, Christensen JP, Winther N, Vingsbo-Lundberg C, et al. Difference in TB10.4 T-cell epitope recognition following immunization with recombinant TB10.4, BCG or infection with Mycobacterium tuberculosis. Eur J Immunol. 2010;40:1342-54.

[57] Davila J, Zhang L, Marrs CF, Durmaz R, Yang Z. Assessment of the genetic diversity of Mycobacterium tuberculosis esxA, esxH, and fbpB genes among clinical isolates and its implication for the future immunization by new tuberculosis subunit vaccines Ag85B-ESAT-6 and Ag85B-TB10.4. J Biomed Biotechnol. 2010;2010:208371.

[58] Rashidian S, Teimourpour R, Meshkat Z. Designing and Construction of a DNA Vaccine Encoding Tb10.4 Gene of Mycobacterium tuberculosis. Iran J Pathol. 2016;11:112-9.
